# The Nuclear Chaperone Nucleophosmin Escorts an Epstein-Barr Virus Nuclear Antigen to Establish Transcriptional Cascades for Latent Infection in Human B Cells

**DOI:** 10.1371/journal.ppat.1003084

**Published:** 2012-12-13

**Authors:** Cheng-Der Liu, Ya-Lin Chen, Yi-Li Min, Bo Zhao, Chi-Ping Cheng, Myung-Soo Kang, Shu-Jun Chiu, Elliott Kieff, Chih-Wen Peng

**Affiliations:** 1 Department of Life Sciences, Tzu-Chi University, Hualien, Taiwan; 2 Department of Medicine and Microbiology and Molecular Genetics, Channing Laboratory, Brigham and Women's Hospital and Harvard Medical School, Boston, Massachusetts, United States of America; University of Wisconsin-Madison School of Medicine and Public Health, United States of America

## Abstract

Epstein-Barr Virus (EBV) is an oncogenic γ-herpesvirus that capably establishes both latent and lytic modes of infection in host cells and causes malignant diseases in humans. Nuclear antigen 2 (EBNA2)-mediated transcription of both cellular and viral genes is essential for the establishment and maintenance of the EBV latency program in B lymphocytes. Here, we employed a protein affinity pull-down and LC-MS/MS analysis to identify nucleophosmin (NPM1) as one of the cellular proteins bound to EBNA2. Additionally, the specific domains that are responsible for protein-protein interactions were characterized as EBNA2 residues 300 to 360 and the oligomerization domain (OD) of NPM1. As in c-MYC, dramatic NPM1 expression was induced in EBV positively infected B cells after three days of viral infection, and both EBNA2 and EBNALP were implicated in the transactivation of the NPM1 promoter. Depletion of NPM1 with the lentivirus-expressed short-hairpin RNAs (shRNAs) effectively abrogated EBNA2-dependent transcription and transformation outgrowth of lymphoblastoid cells. Notably, the ATP-bound state of NPM1 was required to induce assembly of a protein complex containing EBNA2, RBP-Jκ, and NPM1 by stabilizing the interaction of EBNA2 with RBP-Jκ. In a NPM1-knockdown cell line, we demonstrated that an EBNA2-mediated transcription defect was fully restored by the ectopic expression of NPM1. Our findings highlight the essential role of NPM1 in chaperoning EBNA2 onto the latency-associated membrane protein 1 (LMP1) promoters, which is coordinated with the subsequent activation of transcriptional cascades through RBP-Jκ during EBV infection. These data advance our understanding of EBV pathology and further imply that NPM1 can be exploited as a therapeutic target for EBV-associated diseases.

## Author Summary

Epstein-Barr Virus (EBV) infects human B cells to establish a permanent infection in hosts, which can cause diseases ranging from infectious mononucleosis to a broad spectrum of human malignancies. The conversion of human primary B cells into indefinitely proliferating lymphoblastoid cell lines (LCLs) by in vitro EBV infection provides a suitable model for virus-mediated cellular transformation. Epstein-Barr nuclear antigen (EBNA) 2-mediated transcription is essential for the establishment and maintenance of EBV latent infection. In this report, we have extensively explored the mechanism by which EBNA2 activates the latency-specific LMP1 promoter to establish a permanent infection in B cells. We have identified and characterized the protein-protein interaction of EBNA2 with the nuclear shuttle protein nucleophosmin (NPM1) in vivo and in vitro. In particular, we have determined that the expression of NPM1 is promptly induced upon EBV infection and that EBNA2 has a role in activating NPM1 gene expression. Furthermore, we have shown that oligomerized NPM1 is charged by ATP and binds to EBNA2, which is crucial for its ability to stabilize its interaction with the DNA binding protein RBP-Jκ, which is in turn essential for supporting the transcriptional cascades of EBV latent infection. Our findings provide striking evidence to illustrate a new model for understanding EBV pathology.

## Introduction

EBV is a human γ-herpesvirus that infects both epithelial cells and B lymphocytes, and it has been implicated in several human malignancies, including Burkitt's lymphoma (BL), Hodgkin's lymphoma, lymphoproliferative disease in immune-suppressed patients, nasopharyngeal carcinoma (NPC), and some cases of gastric cancer. The primary infection of B lymphocytes by EBV in vitro readily leads to the establishment of immortalized lymphoblastoid cell lines (LCLs) via the expression of a unique set of viral genes, including six EBV nuclear antigens (EBNAs), three latency-associated membrane proteins (LMPs), Bam A rightward transcripts, and small non-coding RNAs [1]. Among the EBV latency gene products, EBNALP, EBNA1, EBNA2, EBNA3A, EBNA3C and LMP1 are critical for LCL cell transformation and maintenance. During the initial stage of B cell infection by EBV, the W promoter (Wp) is exclusively employed to drive the transcription of EBNA2 and EBNALP genes, which are produced by the alternative splicing process. Switches in the usage of Wp to the C promoter (Cp) and activation of Cp by expression of EBNA2 and EBNALP subsequently leads to the concomitant expression of other EBNAs and LMPs that are essential for the latent infection and persistence of EBV {for reviews, see [1]}.

The activation of cellular and viral genes by EBNA2 is coordinated with its biological role in supporting EBV-mediated conversion of resting B cells into LCLs [1]. In general, EBNA2 engages in transcription activation through interactions with cellular DNA-binding proteins, including RBP-Jκ, PU.1, and AUF1, which are tethered to the cognate responsive elements [2], [3], [4], [5]. The C-terminal acidic domain (AD) of EBNA2 recruits the basal transcription machinery, which then performs a major role during transcription activation by forming a transcriptional pre-initiation complex (PIC) [6], [7], [8], [9]. The N-terminus harbors the second activation domain, which plays another role in promoter up-regulation and LCL cell maintenance [10], [11]. Furthermore, the survival motor neuron protein (SMN), co-activators, such as p300/CBP, PCAF, p100 and SKIP1, and the hSWI/SNF chromatin remodeling complex have all been shown to facilitate EBNA2-dependent transcription via interactions with EBNA2 [12], [13], [14], [15], [16], [17]. Although the mechanism by which EBNA2 activates its target genes has been intensively studied over decades, the host factors that are required in addition to the basal transcription machinery and the transcription co-activators that are involved in EBNA2-mediated transcription remain poorly understood.

Nucleophosmin (NPM1, also known as protein B23, NO38, or numatrin) was originally identified as an abundant nuclear phosphoprotein that resides in the nucleolus. It has attracted significant attention for its multifunction capabilities in nuclear shuttling, DNA-histone and nucleosome assembly, biogenesis of ribosomal RNA, regulation of cell growth, DNA repair, cell proliferation, and transformation [18], [19], [20], [21], [22], [23], [24], [25]. The fact that NPM1 is frequently mutated, rearranged, and overexpressed in hematological disorders has led investigators to propose that NPM1 could be a proto-oncogene [21]. By contrast, NPM1 interacts with HDM2 to protect p53 from degradation, and its absence results in mislocalization and protein instability of the tumor suppressor ARF, suggesting an additional role for NPM1 in tumor suppression [26], [27]. At the moment, NPM1 is believed to employ sophisticated mechanisms to engage in oncogenic and tumor suppressor pathways [28].

By acting as a molecular chaperone, NPM1 is able to modulate chromatin assembly and facilitate the DNA binding of the primary transcription factors to their responsive elements, which consequently promotes the transcription of their target genes [22], [25], [29]. In this study, we identified NPM1 as one of the cellular proteins bound to EBNA2 using a protein affinity pull-down assay followed by liquid chromatography-mass spectrometry (LC-MS/MS) analysis. The physical interaction mediated by EBNA2 and NPM1 was extensively characterized, which was proposed as the major determinant of PIC assembly. Finally, we provided striking evidence to show that ATP-charged NPM1 does indeed act as a chaperone to escort EBNA2 to couple with RBP-Jκ at the LMP1 promoter in order to initiate the subsequent EBNA2-dependent transcription, leading to the establishment and maintenance of EBV latency in B cells.

## Results

### NPM1 binds to EBNA2 both in vitro and in vivo

**Figure 1:**
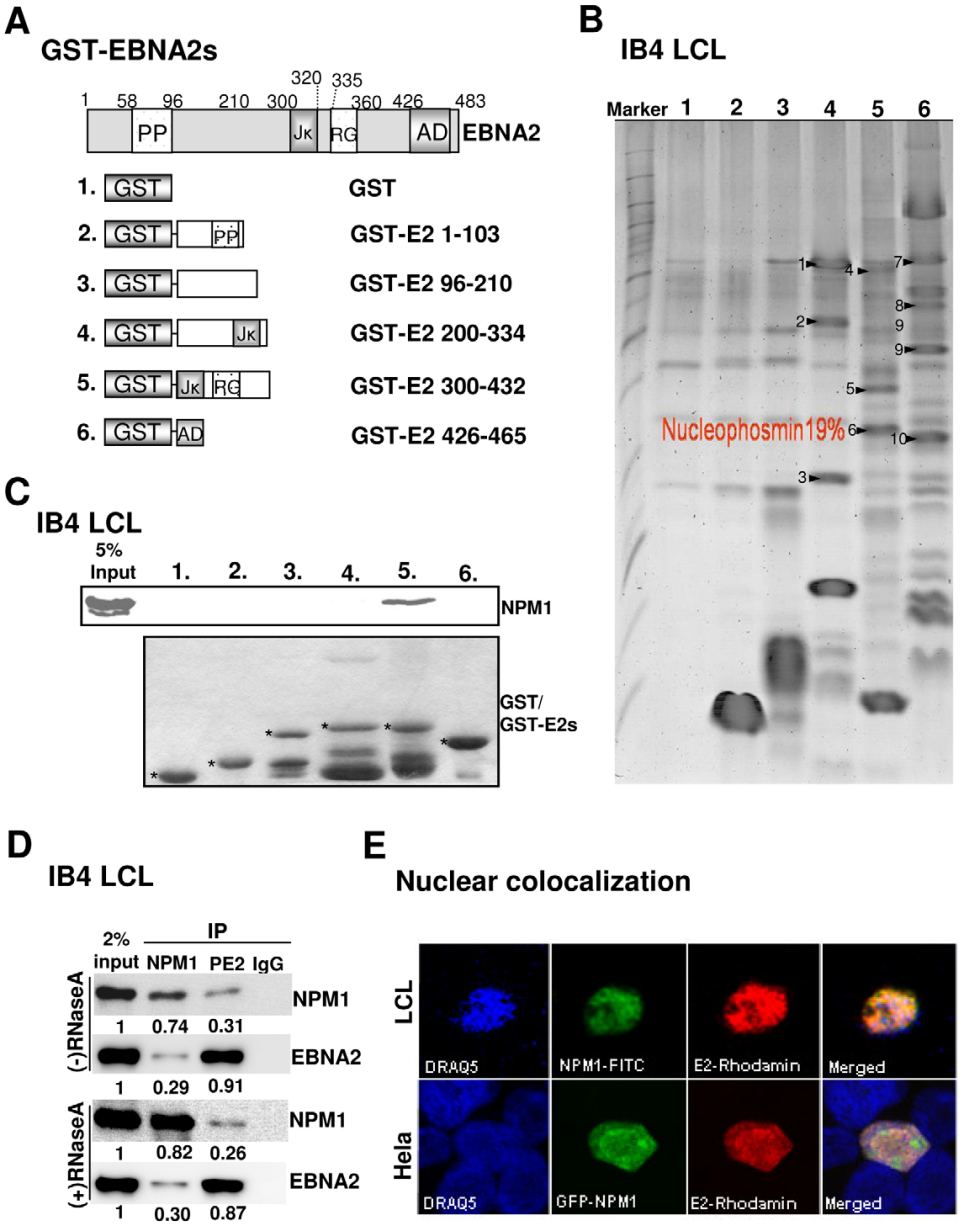
Protein-protein interactions mediated by EBNA2 and NPM1 in vitro and in vivo. A). Schematic depiction of the GST-EBNA2 fusion proteins (GST-E2s). B). IB4 cell lysates were pulled down by similar amounts of each protein bait and resolved on a 4–20% gradient SDS-PAGE gel. Unique gel bands were visualized by staining with Coomassie blue and excised for LC-MS/MS analysis. The NPM1 band is marked with arrowhead 6. C). The samples prepared in (B) were additionally immunoblotted with the NPM1 antibody. The amounts of GST or GST-E2 fusions were determined by Coomassie blue staining, and each is marked with an individual asterisk. D). IB4 cells (10^7^) were subjected to IP analysis using antibodies for EBNA2 (PE2), NPM1 (C-19), or IgG control, and the immunoprecipitated proteins were identified by Western blot with 2% input of EBNA2 and NPM1 as loading controls. The relative expression of each protein was quantified by UN-scan and determined as a percentage of input. E). The immunofluorescent (IF) analyses were carried out using antibodies for EBNA2 (v-C20) and NPM1 (C-19) followed by counterstaining with a goat anti-mouse antibody conjugated to FITC (Green) or a donkey anti-goat antibody conjugated to rhodamine (red). Confocal images for EBNA2 (E2; Red) and NPM1 (Green) from LCL (upper panels) or HeLa cells (bottom panels) that had been co-transfected with EBNA2 and GFP-NPM1 expression vectors are shown. Nuclei were counterstained with DRAQ5 (blue).

To explore the mechanism of EBNA2-mediated transcription in EBV-transformed B cells, we initially aimed to investigate the protein-protein interaction map of EBNA2. We expressed and purified GST and GST fusion proteins with overlapping EBNA2 open reading frames (ORFs) (GST- E2s), including amino acids (aa) 1–103, 96–210, 200–334, 300–432, and 426–465 (AD), which covered almost the entire EBNA2, from *E. coli*. Affinity matrices were used to pull down cellular proteins from IB4 cell lysates (Figure 1 A–C). GST-E2 300–432 pulled down a unique protein band with a molecular weight approximately 33 kD (Figure 1B). LC-MS/MS identified this band as NPM1 with peptide sequences covering 19% of the NPM1 ORF (Figures S1 A and B). Proteins pulled down by the same bait were followed by immunoblotting with the NPM1-specific antibody, which further validated this interaction (Figure 1C).

To characterize the physical interaction mediated by EBNA2 and NPM1 in a physiologically relevant environment, we performed co-immunoprecipitation (co-IP) and immunofluorescence (IF) confocal microscopy analyses. In IB4 LCL, NPM1 and EBNA2 antibodies were used to immunoprecipitate approximately 0.8% of endogenous EBNA2 and NPM1, and the protein complex formed by EBNA2 and NPM1 was not affected by RNaseA treatment (Figure 1D), excluding the possibility that cellular RNAs might act as an intermediate bridge to link the two proteins together. Furthermore, EBNA2 and NPM1 mostly co-localized in the nucleoplasm of the lymphoblastoid cells (Figure 1E, upper panel). HeLa cells transfected with both GFP-NPM1 and EBNA2 expression vectors also showed a pronounced nuclear co-localization (Figure 1E, lower panel), indicating a substantial physical interaction mediated by EBNA2 and NPM1 that took place in vivo.

### The physical interaction of EBNA2 with NPM1 is mediated by EBNA2 aa 300–360 and the N-terminal OD of NPM1

**Figure 2:**
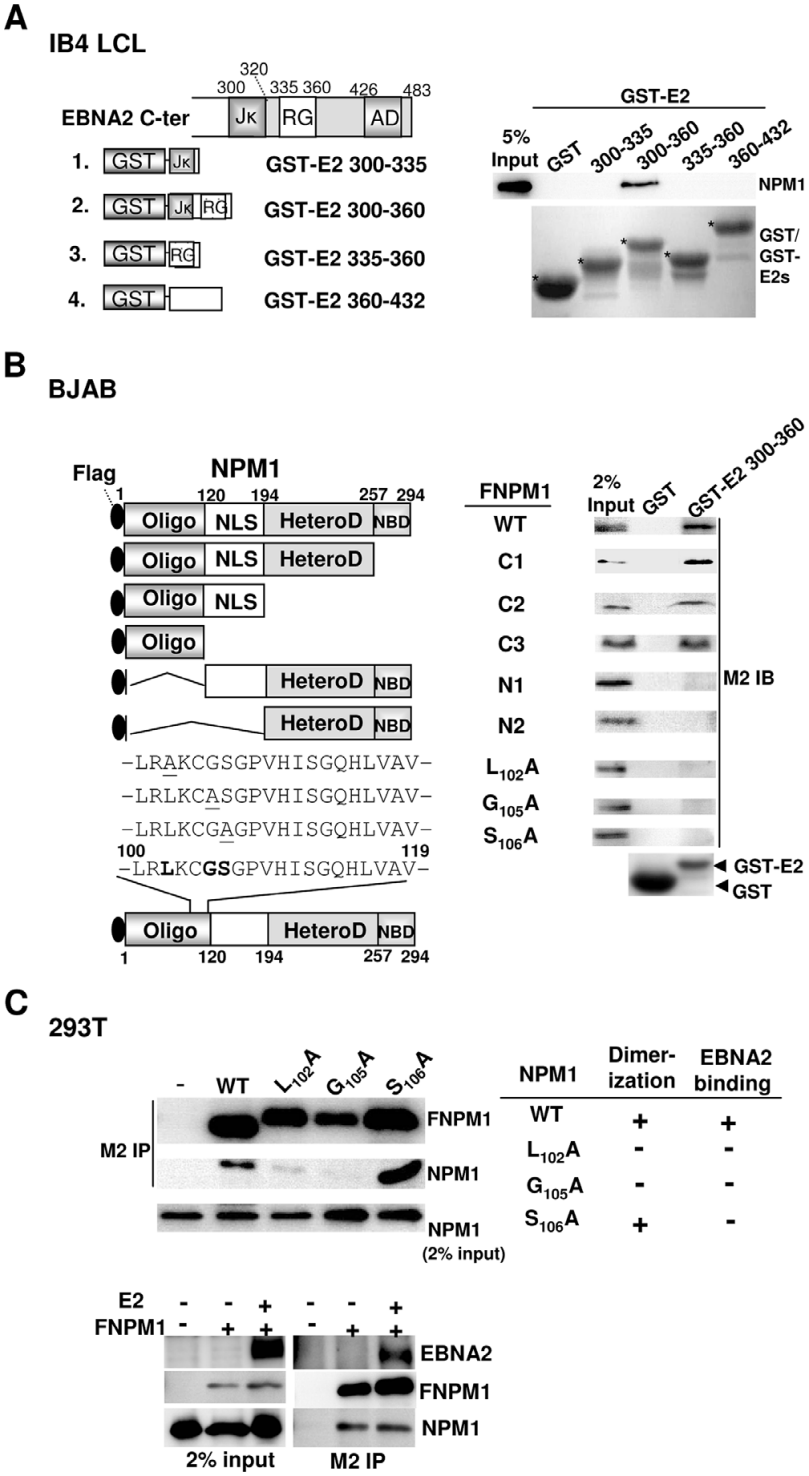
EBNA2 aa 300–360 and the NPM1 N-terminal OD are responsible for the specific physical interaction between the two proteins. A). Schematic diagram of the GST-E2s used in this assay. The Coomassie-blue stained image for GST and GST-E2 fusion proteins (marked with asterisks). The amounts of endogenous NPM1 from the IB4 cell lysate pulled down by the corresponding protein baits were assayed by immunoblot analysis. Five percent input of NPM1 is shown. B). Schematic depiction of the flag-tagged wild type (FNPM1), truncated deletion, and point mutants of NPM1. The conserved amino acid residues are shown in boldface, and the mutated sequences are underlined. BJAB cells (10^7^) transfected with 30 µg of the indicated expression vectors were subjected to protein affinity pull-down assays using GST 300–360 after 24 hours of transfection. The amounts of transfected FNPM1 and other mutants bound to the protein bait were determined by immunoblot with the M2 antibody (a flag-tagged specific antibody). Two percent input of each was used as a loading control. C). 293T cells (2×10^6^) transfected with 3 µg of the FNPM1 or the indicated NPM1 mutants expression plasmids were used for M2-IP analysis after 24 hours of transfection. The interactions mediated by endogenous NPM1 and the transfected FNPM1 or its mutant derivatives were determined (Lower panel). Two percent input of endogenous NPM1 is shown.

To identify the core of the NPM1-binding domain located within EBNA2 aa 300–432, GST and GST-E2 fusions that contain small portions of the EBNA2 ORF, including aa 300–335, 335–360, 300–360, and 360–432, were next used in a protein affinity pull-down assay (Figure 2A). Approximately 2.5% of endogenous NPM1 was pulled down by GST-E2 300–360; however, neither GST nor the remaining GST-E2s was shown to possess any detectable NPM1 binding potency.

GST-E2 300–360 was further utilized as the protein matrix to pull down ectopically expressed flag-tagged NPM1 (FNPM1) and five truncated deletion mutants, including C1 (1–257), C2 (1–194), C3 (1–120), N1 (120–294), and N2 (194–294) [30]. Among the exogenous proteins, including FNPM1 and its mutants, approximately 2% of FNPM1, C1, C2, or C3 was associated with GST-E2 300–360, whereas neither N1 nor N2 was pulled down by the same protein bait (Figure 2B). The N-terminus of NPM1 contains highly conserved amino acid residues that are typically found in members of the nucleophosmin family, which have been implicated in homotypic interactions [23]. In response to this information, the transfected flag-tagged oligomerization-defective mutants L_102_A and G_105_A and another oligomerization-competitive mutant, S_106_A [23], were subjected to additional co-IP and protein affinity pull-down analyses. In each transfection assay, plasmid-expressed FNPM1 or three point mutants were expressed at similar levels and efficiently immunoprecipitated by the flag-specific antibody. Approximately 2% of endogenous NPM1 was co-immunoprecipitated with FNPM1 and S_106_A, while fairly small and barely detectable amounts of this protein were co-immunoprecipitated with L_102_A and G_105_A, respectively (Figure 2C; upper panel). The interaction between exogenous FNPM1 and endogenous NPM1 was not affected by ectopically expressed EBNA2 (Figure 2C; lower panel). Interestingly, our result showed that neither of the ectopically expressed NPM1 mutants was associated with GST-E2 300–360 (Figure 2 B). Taken together, our data reveal that the conserved oligomerization domain (OD) of NPM1 and EBNA2 aa 300–360 mediate the protein-protein interaction between EBNA2 and NPM1.

### NPM1 expression is induced in EBV positively infected B lymphocytes

**Figure 3:**
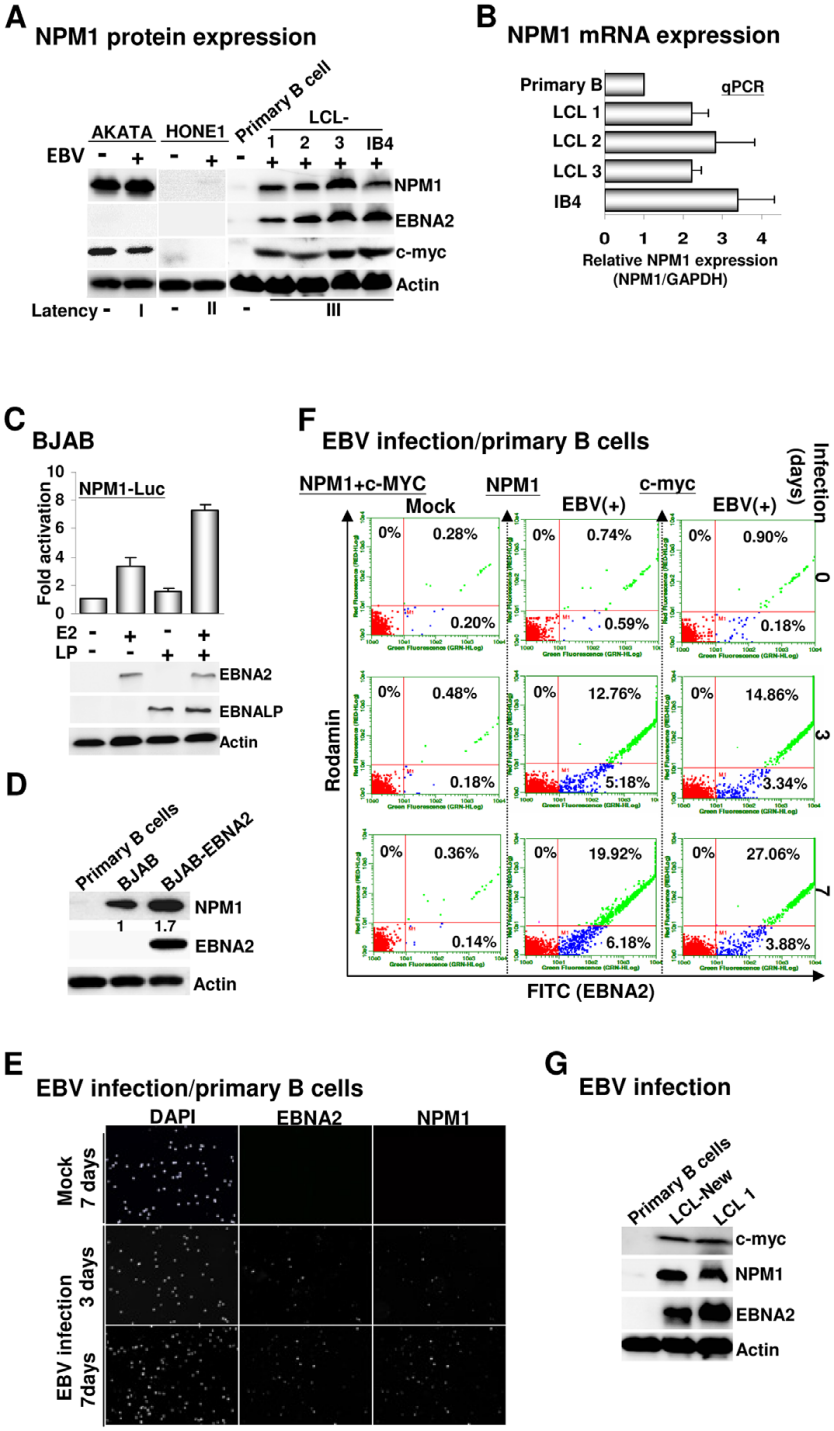
Activation of NPM1 expression is highly associated with EBV infection. A). The immunoblots for NPM1, EBNA2, c-MYC, and actin control from EBV latently infected type I AKATA BL cells, type II HONE1 nasopharyngeal carcinoma cells (NPC), type III LCLs, and EBV negatively infected AKATA, NPC HONE1, and primary B cells. B). The mRNA expression levels of NPM1 in primary B cells and four individual LCLs were determined by quantitative real-time PCR (qPCR), respectively. The amount of NPM1 mRNA is expressed in relation to that of the GAPDH control. C). The effects of EBNA2 and EBNALP on the NPM1-Luc reporter plasmid were determined by the transfection-mediated reporter assay. Error bars represent the standard deviation from the mean for data from at least three independent experiments in this and subsequent figures. The immunoblotting images for transfected EBNA2, EBNALP, and the actin control are shown. D). The expression levels of NPM1, EBNA2, and actin in primary B cells, BJAB, or BJAB-EBNA2 were determined. E–F). Primary B cells (5×10^4^) were infected with EBV or PBS (Mock) and subjected to an IF staining protocol using antibodies for EBNA2 (v-C20), NPM1 (C-19), or c-MYC at 0, 3 or 7 days after infection (dai) followed by a donkey anti-goat antibody conjugated to FITC (Green) or a goat anti-mouse antibody conjugated to rhodamine (red). For mock infection, the Y-axis represented the signaling resulted from a double staining procedure using c-MYC and NPM1 antibodies, whereas the X-axis represented the signaling resulted from an EBNA2 immunostaining protocol. Nuclei were counterstained with DAPI. The immunostained cells were visualized by fluorescent microscopy and quantified by flow cytometry (F). G). The newly established LCL (LCL-New), or previously established LCL (LCL1) and primary B cells were assayed for EBNA2, c-MYC, and actin control expression levels.

To address the possibility that NPM1 has a role in EBV-associated malignancies, the expression levels of NPM1 from an equal amount of BL, NPC, and LCLs cell lysates were assayed by immunoblot analysis. We found that substantial amounts of NPM1 were expressed in AKATA BL cells regardless of EBV status. By contrast, only relatively low levels of NPM1 were observed in EBV-infected NPC cells and the amounts of NPM1 in EBV-negative NPC cells were at a barely detectable level (Figure 3A). In addition to the hallmarks of the EBV latency III program, EBNA2 and its target gene c-MYC, a robust activation of NPM1 gene expression was also detected in four individual LCLs, while none of the proteins (EBNA2, c-MYC and NPM1) was detected in primary B cells. Quantitative real time PCR (qPCR) further identified that the mRNA levels of NPM1 were activated by 2- to 3.5-fold in LCLs in comparison to primary B cells (Figure 3B). A transfected NPM1 reporter plasmid (NPM1-Luc) was consistently induced by EBNA2 to reach 4-fold activation, and EBNALP co-activated the EBNA2 effect to result in 8-fold total activation, which further up-regulated EBNA2-inducing NPM1 promoter activity by 2-fold (Figure 3C). Furthermore, a 1.7-fold increase in the NPM1 protein expression level was detected in BJAB cells that were stably expressing EBNA2 (BJAB-E2) in comparison with the basal level identified in control BJAB cells (Figure 3D). These results indicate that EBNA2 and EBNALP have a major role in activating NPM1 transcription in EBV-transformed B cells.

To verify that the induction of NPM1 in lymphoblastoid cells is directly associated with EBV infection, the expression patterns of NPM1, EBNA2, and c-MYC control in primary B cells were monitored by microscopy or flow cytometry-mediated IF analysis after 0, 3 or 7 days of virus or mock infection. As expected, only the background signals of EBNA2, c-MYC, and NPM1 were detected in mock-infected B cells from 0 to 7 days. EBNA2, the key activator of EBV latent infection, began to be expressed in approximately 18% of B cells at 3 days post-infection (dpi) and increased to 26–30% at 7 dpi (Figures 3 E, F, S2A–B, and Table S1). The immunostained spots of c-MYC appeared in 14.86% of B cells at 3 dpi and increased to 27.06% at 7 dpi (Figures 3F, S2B, and Table S1). Similarly, NPM1-positive cells were observed in 12.76% of B cells and increased to 19.92% at 7 dpi (Figures 3 E, F, S2A, and Table S1). Notably, the c-MYC- or NPM1-expressing cells were all detected in EBV positively infected B cells, indicating an absolute dependence of c-MYC and NPM1 expression on EBV infection. Finally, we showed consistent expression of both c-MYC and NPM1 in a newly established LCL rather than in primary B cells (Figures 3G and S2C), which further validated the hypothesis that NPM1 expression induction was highly associated with EBV infection.

### NPM1 is essential for EBNA2-mediated transcription

**Figure 4:**
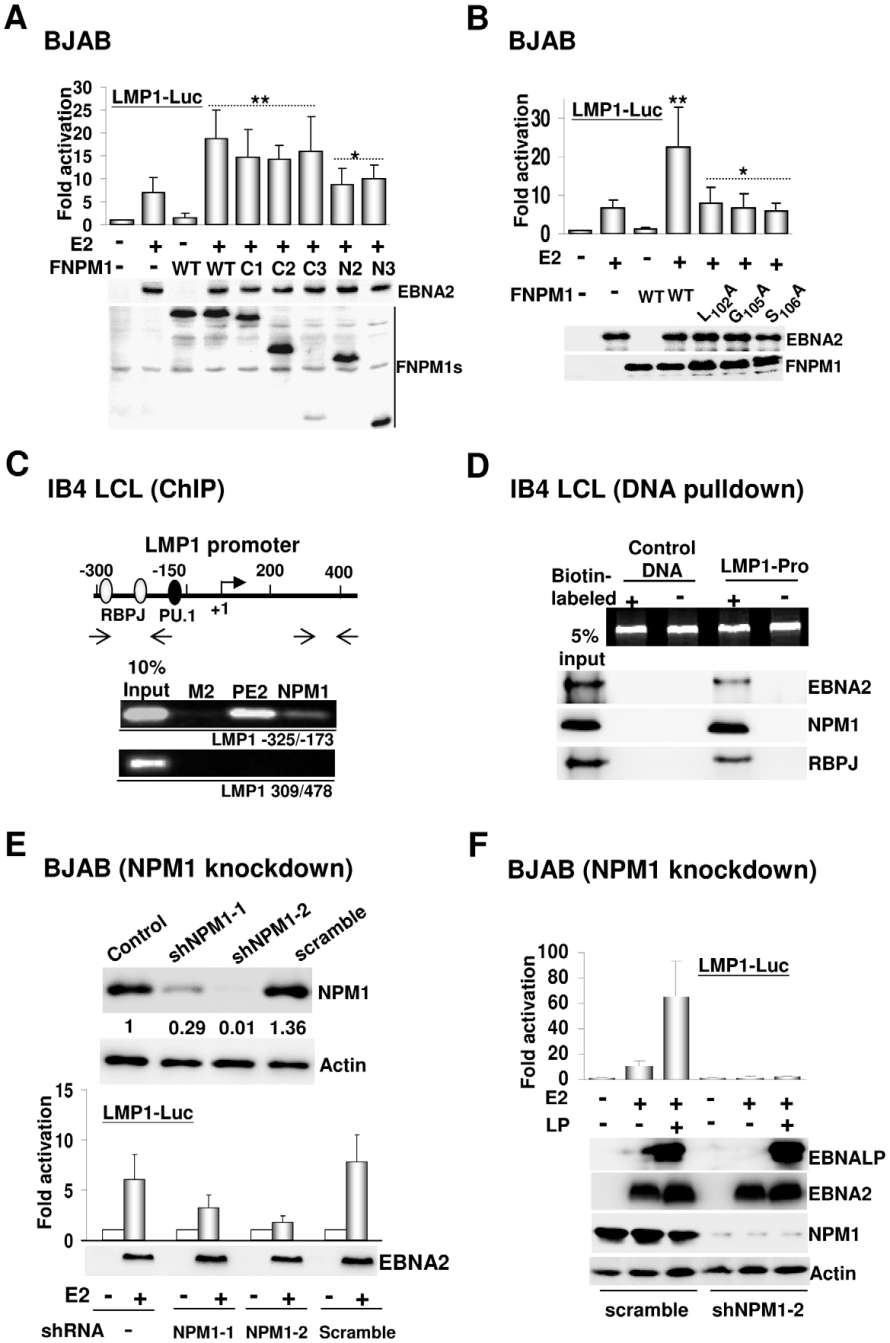
NPM1 is essential for EBNA2-inducing LMP1 promoter activity. A–B). The transfected FNPM1 and the indicated NPM1 mutants were assayed for their effects on EBNA2-inducing LMP1-Luc activity after 24 hours of transfection. Immunoblots for ectopically expressed EBNA2 and FNPM1s in this assay are shown. The resulting activity from each assay was represented as the mean ± standard deviation for the data from three individual experiments.^*^P value>0.05 vs. control, and ^**^P value <0.05. C). A schematic depiction of the cognate responsive elements located at the LMP1 promoter. Promoter occupancy of EBNA2, NPM1, or the control flag-epitope antibody was identified by ChIP analysis using the corresponding antibodies, i.e., PE2 for EBNA2, 5E3 for NPM1, and M2 for flag, and then quantified by PCR. D). Proteins bound to the corresponding DNA fragments were identified by the streptavidin-agarose mediated pull-down assay followed by immunoblot analysis. Ten percent input of the DNA fragments and 5% of each protein are shown. E). The EBNA2-inducing LMP1-Luc activity in each NPM1 knockdown cell line was determined by the transfection-mediated reporter assay. The expression levels of the transfected EBNA2 in the control and shRNA transduced cell lines were verified by Western blot. F). EBNALP co-activation with EBNA2-dependent transcription of LMP1-Luc was monitored in scrambled shRNA and shNPM1-2 expressing BJAB cells. The immunoblots for the transfected EBNA2, EBNALP, endogenous NPM1, and the actin control are shown.

The fact that NPM1 has a role in transcription up-regulation via its associations with histone proteins inspired us to investigate whether NPM1 is involved in EBNA2-mediated transcription [22]. In transfection-mediated EBNA2-dependent transcription assays, the expression levels of EBNA2 were not affected by the co-transfected FNPM1 or any other NPM1 mutants (Figures 4 A and B). EBNA2-inducing LMP1-Luc activity was maintained at 8- to 12-fold activation. We showed that neither the transfected FNPM1 nor any of the NPM1 mutants could affect the basal activity produced by the LMP1-Luc reporter plasmid alone. Ectopic expression of FNPM1 readily augmented the EBNA2-dependent LMP1-Luc activity to reach an average of 20- to 25-fold total activation, which induced a 2.5-fold co-activating effect (Figure 4A). Similarly, the transfected C1, C2, or C3 elicited a 2.5-fold increase over the intrinsic EBNA2-inducing LMP1-Luc activity. By contrast, neither ectopically expressed N1 nor N2 was able to co-stimulate with EBNA2, indicating that NPM1 N-terminal aa 1–120 contributed the major transcriptional up-regulating effect. Following the loss of the EBNA2 binding affinities found in the NPM1 mutants L_102_A, G_105_A, and S_106_A, we next investigated their potency in co-stimulation with EBNA2. Although plasmid-expressed FNPM1 co-stimulated with EBNA2 by 2.5-fold, we found that all transfected NPM1 mutants lost the ability to up-regulate the EBNA2-inducing LMP1-Luc activity (Figure 4B). These data reveal that NPM1-mediated self-association and interaction with EBNA2 are the prerequisite requirements for co-stimulation with EBNA2.

Both ChIP and streptavidin-agarose-mediated DNA pull-down analyses were further employed to gauge the abundance of NPM1 at the EBV latency-associated LMP1 promoter [31]. The amounts of EBNA2 and NPM1 bound to this cognate promoter were identified as 6% and 2% of the input DNA by PCR (Figure 4C). In addition, the biotin-labeled LMP1 DNA pulled down approximately 2% of endogenous EBNA2 and RBP-Jκ and 5% of endogenous NPM1 (Figure 4D), whereas neither of the proteins was precipitated by the biotin-labeled or non-labeled control DNA and the non-labeled LMP1 DNA. We then sought to explore whether NPM1 is essential for EBNA2-mediated transcription. The EBNA2-inducing LMP1-Luc activity was assayed in BJAB cells in which endogenous NPM1 was stably silenced by the lentivirus-expressed NPM1 shRNA (shNPM1-1 or shNPM1-2), or scrambled shRNA. BJAB cell lines that stably expressed each shRNA, which were designated as BJAB-shNPM1-1, BJAB-shNPM1-2, and BJAB-scrambled, or control BJAB cells were subjected to transfection-mediated reporter assays. The expression levels of the actin control were not affected by any of the transduced shRNAs, so the amounts of NPM1 detected in each cell line was expressed in relation to that of the control BJAB cells, giving 71%, 99%, and 0% NPM1 knockdown efficiency in the BJAB- shNPM1-1, shNPM1-2, and scrambled cell lines, respectively (Figure 4E). With similar expression levels resulted by the transfected EBNA2, the EBNA2-inducing LMP1-Luc activity could reach 6- and 8-fold activation in the control and BJAB-scrambled cell lines, whereas this EBNA2-dependent activity was reduced down to 3- and 1.5-fold activation in the BJAB-shNPM1-1 and BJAB-shNPM1-2 cell lines. In addition, the defect of EBNA2-mediated transcription caused by the stable NPM1 depletion could not be restored by the co-transfected EBNALP (Figure 4F). Our data reveal the dominant importance of NPM1 in EBNA2-dependent transcription.

### NPM1 stabilizes the interaction of EBNA2 with RBP-Jκ to induce the formation of a putative PIC

**Figure 5:**
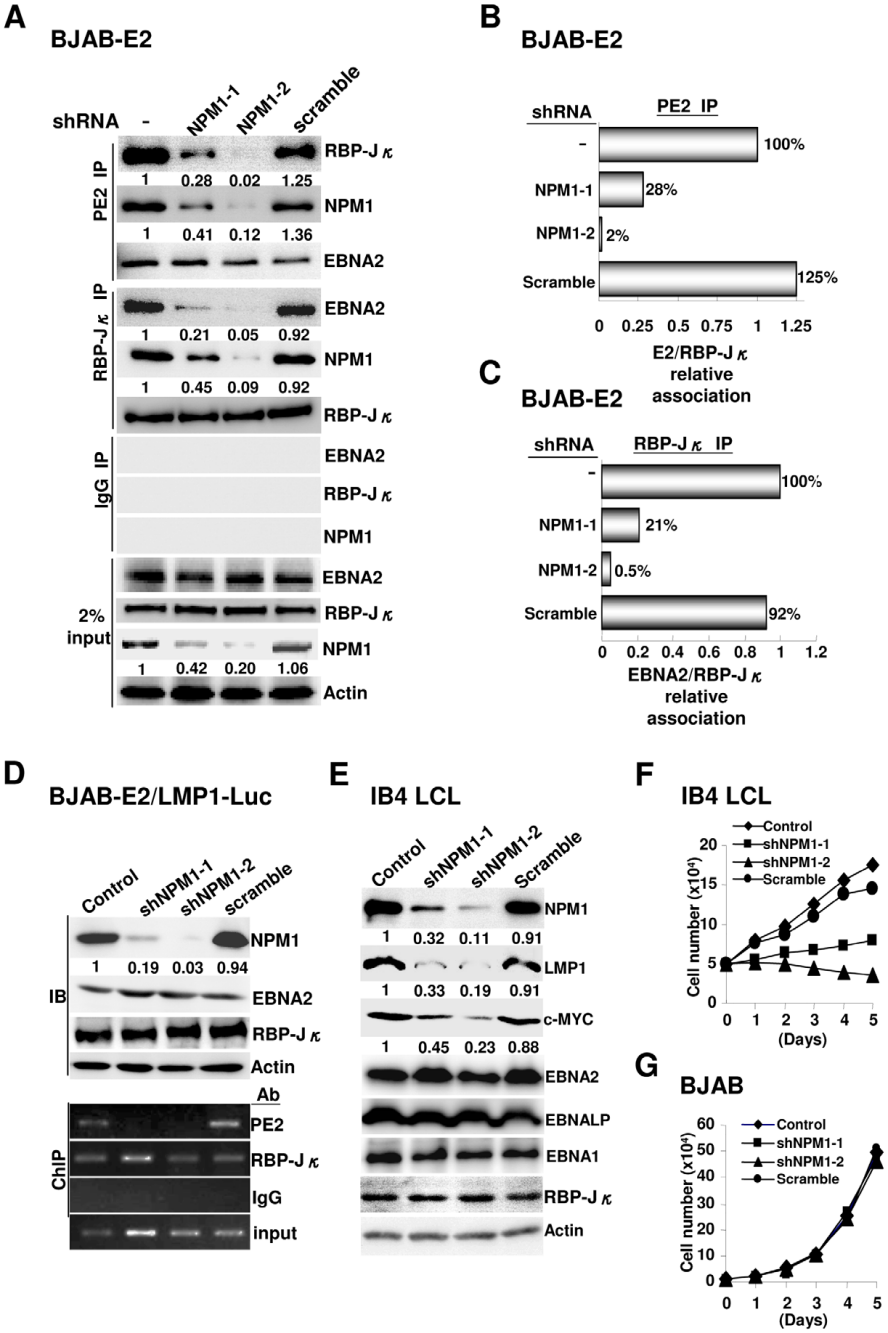
NPM1-mediated EBNA2 recruitment onto the LMP1 promoter is essential for the maintenance of lymphoblastoid cells. A). NPM1-depletion, scrambled shRNA expression, or control BJAB-E2 cells were subjected to IP analysis using antibodies for EBNA2, RBP-Jκ, and IgG control, respectively. The expression levels of EBNA2, RBP-Jκ, NPM1, and actin control in each cell line were determined by Western blot. The amounts of EBNA2, BRP-Jκ, and NPM1 contained in each immunoprecipitated protein complex and 2% input of each protein are shown. The immunoblotted images were further quantified using UN-scan. The amounts of the indicated proteins identified in the NPM1 or scrambled shRNA knockdown cell lines are expressed relative to the protein levels observed in the control cells. The relative amount of each protein is shown at the bottom of the image. B–C). The summary of data from A (the first and fourth panels, respectively) is converted as a histogram showing the relative amounts of co-immunoprecipitated RBP-Jκ (B) or EBNA2 (C) from the indicated BJAB-E2 cell lines, which represents the magnitude of EBNA2 and RBP-Jκ association. D). BJAB-E2/LMP1-Luc cells transduced with the lentivirus-expressed NPM1 shRNAs or scrambled shRNA were subjected to a ChIP assay. Immunoblots for the proteins of interest and actin control from each cell line are shown. The promoter occupancy of each protein was quantified by PCR. E). The expression levels of the indicated proteins in each NPM1-depleted or IB4 LCL control were assayed by immune blot. F). IB4 LCL and its derivatives described in (E) were measured for growth over five consecutive days. G). The growth curves represented for the shRNA knockdown and control BJAB cells are shown.

The fact that NPM1 has been shown to enhance chromatin transcription by acting as a histone chaperone [22] inspired us to hypothesize that NPM1 is involved in recruiting EBNA2 onto the EBV latency-associated promoters. BJAB-E2 cells transduced with shRNAs, as described in Figure 4E, were used to monitor the changes in protein-protein interactions mediated by EBNA2 and RBP-Jκ by co-IP analysis. Endogenous EBNA2, RBP-Jκ, and actin control were expressed at similar levels, and both RBP-Jκ and EBNA2 were efficiently immunoprecipitated by their corresponding antibodies from each cell lysate (Figure 5A). Our result showed that a 58% knockdown of NPM1 decreased the amounts of co-immunoprecipitated RBP-Jκ and EBNA2 to 28% and 21%, whereas 80% depletion of NPM1 dissociated almost 100% of EBNA2 and RBP-Jκ from the immunoprecipitated protein complex. By contrast, the magnitude of the physical interaction between EBNA2 and RBP-Jκ that was identified in the scrambled shRNA-expressing cells remained similar to the level detected in control BJAB-E2 (Figures 5A–C). Moreover, our results also indicated that NPM1 could associate with RBP-Jκ through its N-terminal OD (Figures 5A and S3), which further suggested that NPM1 acted as a connecting bridge to stabilize the interaction between EBNA2 and RBP-Jκ.

To verify that NPM1 escorted EBNA2 to form a complex with RBP-Jκ at the EBV latency-specific LMP1 promoter, we next performed a ChIP assay using EBNA2 and LMP1-Luc reporter co-expressing BJAB cells (BJAB-E2/LMP1-Luc) that had been stably transduced with shRNAs as described in Figure 4E. Lentivirus-mediated expression of shNPM1-1 and shNPM1-2 effectively depleted endogenous NPM1 by 81% and 97%, while the scrambled shRNA remained ineffective in this cell line. Although EBNA2, RBP-Jκ, and actin were expressed at similar levels in the control and in all shRNA-transduced BJAB-E2/LMP1-Luc cell lines, NPM1 deficiency caused a nearly complete dissociation of EBNA2 from the LMP1 promoter (Figure 5D). By contrast, the promoter occupancy of RBP-Jκ was not altered by NPM1 depletion. Our results suggest that NPM1 has a role in the formation of a stable PIC.

### The involvement of NPM1 in EBNA2-mediated transcription is coordinated with the cell maintenance of EBV-transformed lymphoblastoid cells

To verify that the involvement of NPM1 in EBNA2-dependent transcription is coordinated with the subsequent maintenance of lymphoblastoid cells, the expression levels of EBNA2 target genes and cell proliferation were assayed using IB4 LCLs that had been stably transduced with each type of shRNA as described elsewhere in this article. In addition to actin, we found that the expression levels of EBNAs, including EBNA2, EBNALP and EBNA1, and RBP-Jκ in the control cell line were similar to the levels of each protein identified in either the NPM1 shRNA or the scrambled shRNA expressing IB4 LCLs. As expected, the EBNA2 target genes, such as c-MYC and LMP1, were expressed in a way that was inversely correlated with the NPM1 knockdown efficiency (Figure 5E). Although cell growth was slightly impaired by the transduced scrambled shRNA, it remained similar to the phenotype caused by the control IB4 LCL. Strikingly, the cell proliferation of IB4 cells was halted when only 32% of the NPM1 expression was retained, whereas these cells died over time when an 89% knockdown efficiency of NPM1 was reached (Figure 5F). Similar experiments were also conducted using the BJAB cell line and its derivatives as described in Figure 4E. We found that the patterns of cell growth were not altered by NPM1 depletion in the context of BJAB cells (Figure 5G). These data strongly support the hypothesis that NPM1-assisted EBNA2-dependent transcription is linked to the concomitant maintenance of EBV-transformed B cells.

### The ATP-bound state of NPM1 associates with EBNA2 and RBP-Jκ to form the putative PIC

**Figure 6:**
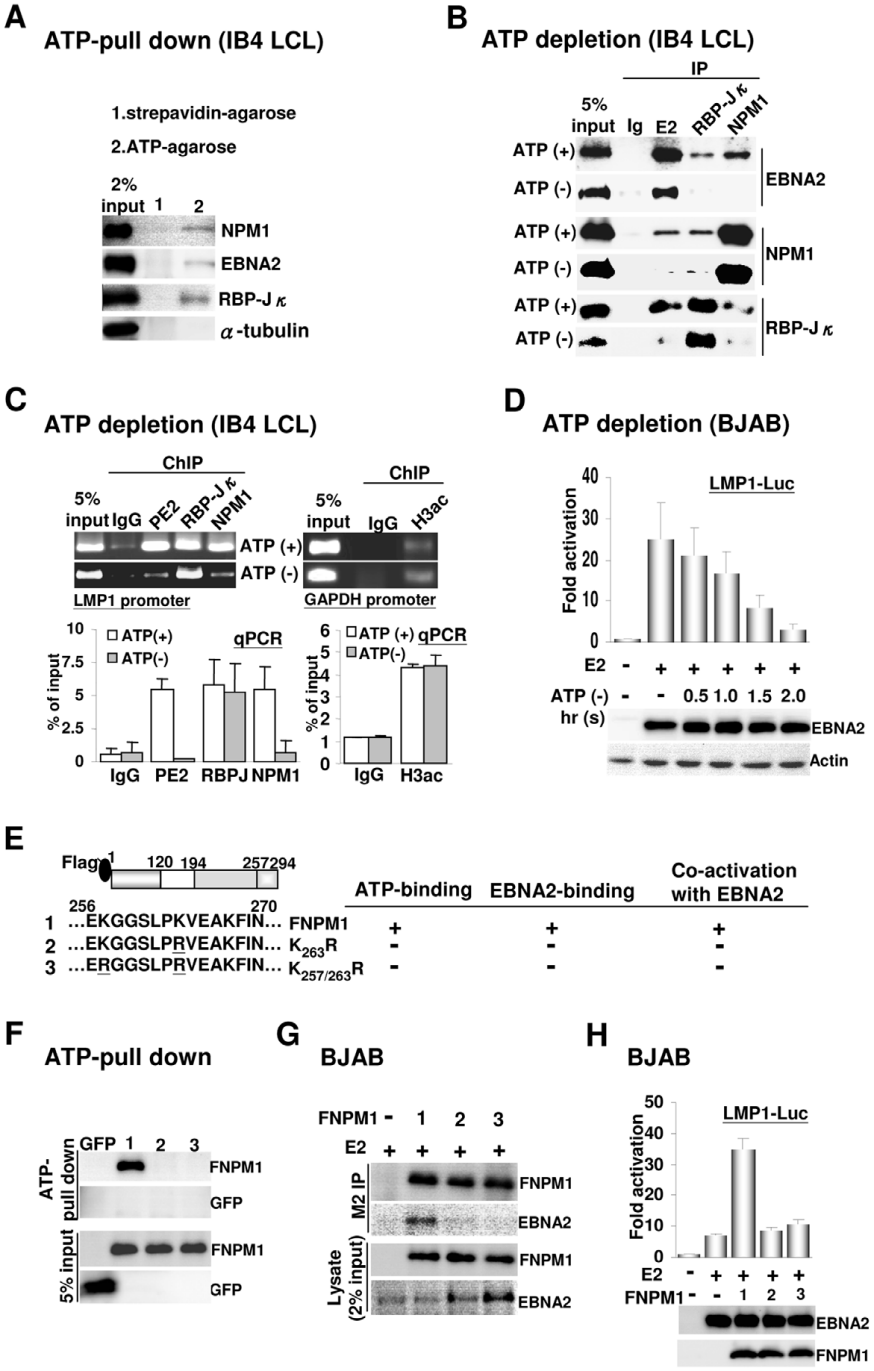
ATP-charged NPM1 induces the assembly of the putative PIC. A). Proteins bound to ATP or control streptavidin-agarose were assayed by immune blot. Two percent input of each protein is shown. B–C). IB4 cells treated with or without ATP depletion were subjected to IP and ChIP analyses exactly as described in Figure 5A and 5D. The immunoblots for the immunoprecipitated, co-immunoprecipitated proteins, and 5% input of the proteins for each assay are shown. The promoter occupancy of each protein was quantified by both PCR and qPCR. Data from qPCR were formulated as a percentage of the input DNA. The abundance of H3ac was assayed for the GAPDH promoter. D). EBNA2-inducing LMP1-Luc reporter activity in BJAB cells that were treated with ATP-depletion over different time courses are shown. The expression levels of the transfected EBNA2 and the actin internal control at different time points were determined. E–F). A schematic representation of the ATP-binding mutants, K_263_R and K_257/263_R. 293T cells transfected with the expression plasmid of FNPM1, FNPM1 mutants, or GFP were used for an ATP-agarose-mediated pull-down assay followed by an immune blot with M2 or GFP antibody. Five percent input of GFP and FNPM1s are shown. G). The interaction between EBNA2 and FNPM1, or each FNPM1 mutant, was identified by co-IP and Western blot. Two percent input of EBNA2 and FNPM1s are shown. H). The up-regulating effects caused by the transfected FNPM1 or two ATP binding mutants on EBNA2-dependent transcription are shown. The expression levels of ectopically expressed EBNA2 and FNPM1s in each assay were determined.

To assess the possibility that the ATP-bound state of NPM1 [32], [33] is involved in the PIC formation, the amounts of EBNA2, RBP-Jκ, and NPM1 from IB4 cell lysates that were precipitated by ATP or streptavidin-agarose were identified by immunoblot analysis. Except for α-tubulin, we showed that approximately 0.4% of endogenous EBNA2, RBP-Jκ, and NPM1 was pulled down by ATP-agarose. By contrast, neither of the proteins was precipitated by streptavidin-agarose (Figure 6A). The ability to form a protein complex containing EBNA2, RBP-Jκ, and NPM1 in IB4 cells was then monitored by co-IP assay after an ATP-depletion protocol. Strikingly, although ATP-depletion partially reduced the EBNA2 and BBP-Jκ expression levels, it vigorously dissociated each protein from the complex. By contrast, the stable protein complex remained in its original form in the control IB4 cells (Figure 6B).

A ChIP assay was carried out to further verify that ATP-depletion could impair the promoter occupancy of EBNA2, RBP-Jκ, and NPM1. In normal IB4 cells, a substantial level of occupancy of each protein at the LMP1 promoter was identified by both PCR and qPCR, which showed an average from 5% to 6% of the input DNA (Figures 6C and S4A). The abundance of promoter-bound RBP-Jκ was not altered while both EBNA2 and NPM1 were almost completely dissociated from the LMP1 promoter in ATP-deficient cells. In addition, our result revealed that the amount of acetylated H3 (H3ac) at the control GAPDH promoter was not affected by ATP-depletion, accounting for 4% of the input DNA (Figures 6C and S4B–C). To confirm that ATP-depletion should consequently lead to the abrogation of EBNA2-dependent transcription, the EBNA2-inducing LMP1-Luc activity was assayed in BJAB cells with 0 to 2 hours of ATP-depletion. Although the expression levels of the transfected EBNA2 and endogenous actin were not altered within the ATP-depletion time frame, a progressive loss of EBNA2-inducing activity was detected upon ATP-depletion in a time-dependent manner (Figure 6D). On the other hand, we demonstrated that the intrinsic activity of the transfected SV40-Luc reporter plasmid was not affected by ATP-depletion (Figure S4C).

To exclude the possibility that our findings could have resulted from the nonspecific effects caused by cell ATP-deficiency, the plasmids of two ATP-binding mutants, K_263_R and K_257/263_R [32], were used to confirm that the ATP-bound state of NPM1 is indeed required for the putative PIC formation following transfection-mediated ATP-agarose pull-down, Co-IP, and reporter assays. Our result showed that 2% of the transfected FNPM1 and three previously identified EBNA2 binding mutants L_102_A, G_105_A, and S_106_A (Figure 2B) were efficiently pulled down by ATP-agarose, whereas plasmid-expressed GFP, K_263_R and K_257/263_R did not possess any detectable ATP-binding affinity (Figures 6E–F; S4E). The co-IP assay indicated that both ATP-binding mutants lost the ability to associate with EBNA2, while the transfected FNPM1 remained effectively bound to EBNA2 (Figure 6G). Furthermore, ectopic expression of the ATP-binding mutants did not cause any co-stimulating effects on the EBNA2-inducing LMP1-Luc activity, while the transfected FNPM1 vigorously augmented this EBNA2-dependent activity by 4-fold (Figure 6H). Altogether, these results suggest that NPM1 needs to be in an ATP-bound state to induce the assembly of the PIC.

### NPM1 binds to EBNA2 and recruits it to the latency-specific LMP1 promoter

**Figure 7:**
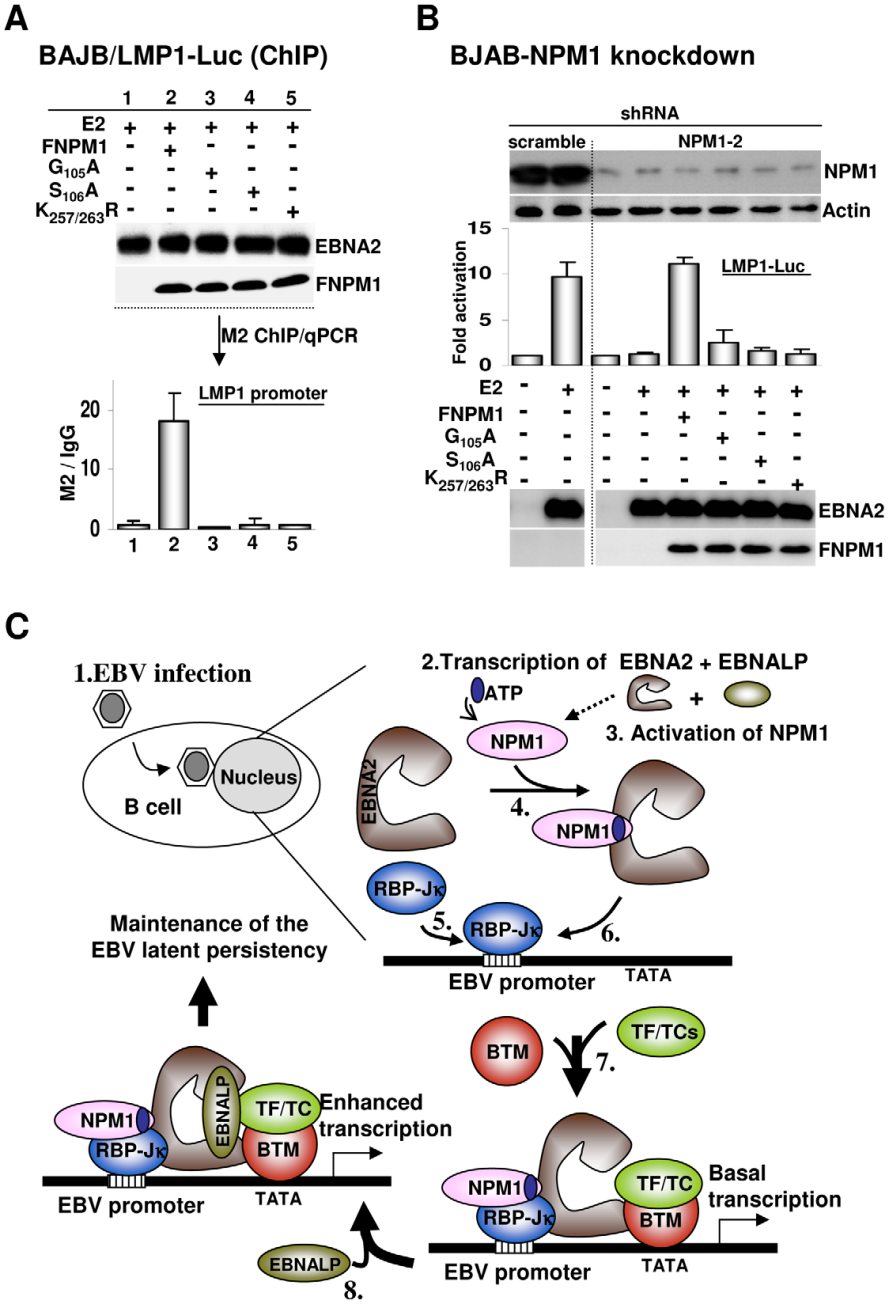
NPM1 binds to EBNA2 and recruits it to the EBV latency-specific LMP1 promoter. A). BJAB-LMP1-Luc cells co-transfected with the plasmids of EBNA2 and FNPM1 or EBNA2 binding mutants were subjected to ChIP assay using M2 antibody or control IgG. The immunoblots for the transfected EBNA2 or FNPM1s are shown. Promoter occupancy of FNPM1 or other mutants was quantified by qPCR. In each case, the data from M2 ChIP were normalized to the background level that was identified for IgG ChIP. B). The transfected FNPM1 and each FNPM1 mutant were used to rescue the defect in EBNA2-dependent transcription of NPM1 knockdown cells. The intrinsic EBNA2-inducing LMP1-Luc activity observed in BJAB-scrambled cells was used as a positive control. The expression levels of the transfected plasmids, endogenous NPM1, and actin control for each assay are shown. C). A new version of the working model proposed for EBNA2-dependent transcription in EBV immortalized B lymphocytes.

Finally, we aimed to provide direct evidence supporting the idea that the recruitment of EBNA2 to the latency-specific LMP1 promoter relies on its interaction with NPM1. To this end, the transfected FNPM1 and three types of EBNA2 binding mutants, including G_105_A, S_106_A, and K_257/263_R, were monitored for their ability to form a complex with EBNA2 at the LMP1 promoter in BJAB/LMP1-Luc cells, respectively. The co-transfected EBNA2 and FNPM1, or its mutant derivatives, were expressed at similar levels (Figure 7A). ChIP assays identified the accumulation of FNPM1 at the LMP1 promoter at approximately 20-fold above the background level found in the IgG control. In contrast, the amount of promoter occupancy identified for each transfected EBNA2-binding mutant was similar to that of the IgG control (Figures 7A and S5). We noted that shNPM1-2 suppressed NPM1 mRNA by targeting its 3′UTR, which allowed us to carry out the plasmid-mediated rescue experiment for the EBNA2-dependent transcription assay in the context of BJAB-shNPM1-2 cell line. The co-transfected EBNA2, FNPM1, and three EBNA2 binding mutants were expressed at similar levels. A complete loss of the EBNA2-inducing activity of LMP1-Luc in BJAB-shNPM1-2 cells was successfully restored by the co-transfected FNPM from the background level to 10-fold activation, which was comparable to the EBNA2-dependent transcriptional activity detected in BJAB-scrambled cells. In comparison, neither of the transfected EBNA2 binding mutants was able to rescue the EBNA2-dependent transcription defect of LMP1-Luc in BJAB-shNPM1-2 cells (Figure 7B). These data imply that the ability of NPM1 to bind to EBNA2 is the most important factor that determines the EBNA2 abundance at its target promoter.

## Discussion

Due to a lack of intrinsic DNA binding activity, the stable association of EBNA2 with RBP-Jκ has been characterized in each EBNA2 target promoter, although the importance of the PU.1 or AUF1 sites in the LMP1 promoter argues that the RBP-Jκ sites are insufficient to support all EBNA2-dependent transcription [1]. The potent interactions with both RBP-Jκ and SKIP that are mediated by the EBNA2 internal region aa 280–337 has long been considered to be a direct force that induces the formation of the PIC [2], [13]. However, the mechanistic basis for this scenario has yet to be elucidated. In this study, we identified the domain that corresponds to EBNA2 aa 300–360 as the NPM1 binding module, which contains a partial overlapping region within the RBP-Jκ binding domain. Oligomerization mediated by the N-terminal region aa 1–120 of NPM1 is known to be coordinated with its major chaperone activity, while the ATP-bound state of NPM1 depends on the sumoylation of the K263 residue, which was previously implicated in protein stability, nuclear distribution, and cell proliferation [32], [34]. Our data reveal that not only the N-terminal OD of NPM1 but also the oligomerized and ATP-bound states of NPM1 are required for maintaining the specific interaction with EBNA2.

The sophisticated functional profiles of NPM1 documented so far have led to the difficulties in the delineation of its biological picture. Nevertheless, the overexpression of NPM1 is found in many types of solid human tumors, including tumors of the thyroid, brain, liver, and prostate [34]. Induction of NPM1 by mitotic agent treatments in B or T lymphocytes further echoes its involvement in mitotic progression and cell proliferation [19], [35]. Moreover, NPM1 has been identified as one of the c-MYC target genes, and it potentially enhances c-MYC-induced hyperproliferation and transformation via a direct protein-protein interaction [20], [36]. Accordingly, we observed a pronounced induction of both c-MYC and NPM1 in EBV positively infected B lymphocytes throughout the duration of viral infection. This finding provides a more comprehensive understanding of why c-MYC is one of the major determinants of the EBV latency III program maintenance [37], [38]. Our results at least partially indicate that EBNA2 and EBNALP play a role in the transactivation of NPM1, and this effect could possibly be further augmented by the subsequent expression of c-MYC. Additionally, the high levels of NPM1 observed in EBV negatively infected AKATA or BJAB BL cells suggest that proliferating B cells constitute an additional means to induce NPM1 expression in the absence of an EBV infection. This assumption can also explain the observation that the NPM1-Luc reporter activity induced by EBNA2 and EBNALP in BJAB BL cells or the overexpressed level of enodogenous NPM1 in the EBNA2 stably expressing BJAB cell line was simply shown as an intermediate phenotype.

The main breakthrough of this study is that ATP-charged NPM1 functions as a connecting bridge that links EBNA2 and RBP-Jκ together to form a complex at the EBV latency-specific promoter LMP1, which implies that ATP-bound state of NPM1 is involved in the formation of a stable PIC. In the absence of either NPM1 or ATP, EBNA2 no longer binds to RBP-Jκ and will dissociate from the LMP1 promoter, while the DNA-binding potency of RBP-Jκ remains unaltered. Thus, the major contribution of NPM1 is that it acts as a chaperone to escort EBNA2 to its target genes. Our findings fill in missing parts of the EBNA2-dependent transcription model, which have been lacking from previous studies. Because neither NPM1 nor EBNA2 possesses bona-fide DNA-binding potency, the DNA-binding protein RBP-Jκ emerges as their intermediate instead. Nevertheless, EBNA2 alone remains insufficient in maintaining the interaction with RBP-Jκ and yet requires a prerequisite binding with NPM1 to stabilize the subsequent interaction with RBP-Jκ. In lymphoblastoid cells, the dominant importance of NPM1 in EBNA2-dependent transcription further supports this proposed scenario. Among the viral latent proteins that are involved in EBV-mediated immortalization of B cells, the expression of LMP1 appears to be highly dependent on EBNA2. As in the EBNA2 target gene c-MYC, an enormous reduction in LMP1 expression was observed in NPM1 knockdown lymphoblastoid cells, while the expression levels of the remaining EBNAs, including EBNA1, EBNA2, and EBNALP, RBP-Jκ and the actin control were not altered. Most importantly, the robust impairments of cell growth caused by NPM1 knockdown in IB4 LCL emphasize the conspicuous biological role of NPM1 in EBV latent infection.

According to our progress in this study, we have constructed the following new model for the EBNA2-dependent transcriptional cascades that are activated in EBV-immortalized B cells (Figure 7C): (1) Entry of EBV into B cells occurs at the initial stage of viral infection. (2) EBNA2 and EBNALP are transcribed via activation of Wp. (3) Activation of NPM1 expression and other EBNA2 and EBNALP-dependent genes, such as c-MYC, CD21, and CD23. (4) Oligomerized NPM1 is charged by ATP to form the ATP-bound state (which is the active form of NPM1). (5) RBP-Jκ is recruited to the binding sites that are located in the EBNA2 target promoters. (6) The active form of NPM1 binds to EBNA2, escorts it to form a complex with RBP-Jκ, and subsequently induces the assembly of a PIC. (7) The EBNA2 C-terminal AD mediates the recruitment of the basal transcription machinery (BTM), transcription factors (TFs), and transcription cofactors (TCs). (8) EBNALP binds to EBNA2 and augments EBNA2-dependent transcription. (9) Expression of cellular and viral genes that are essential for the establishment and maintenance of the EBV latent infection occur.

The involvement of NPM1 in viral replication has been documented in hepatitis delta virus, Japanese encephalitis virus, and adenovirus [39], [40], [41]. Until recently, NPM1 phosphorylation by v-cyclin-CDK6 was thought to be a critical event that was linked to KSHV latency [42]. In addition, the acetylation of NPM1 was further implicated in viral transactivation upon HIV infection [43]. Accumulated evidence implies that NPM1 seems to have co-evolved with the human viruses to facilitate diverse virus infection processes within the nucleus. The fact that EBNA2 mimics an activated Notch receptor to drive transcription through RBP-Jκ during B cell immortalization [44] suggests the possibility that NPM1 represents a prototype nuclear chaperone that is involved in Notch-dependent transcription, although this hypothesis has yet to be confirmed. Our studies open a new window for exploring the molecular models of EBNA2 and Notch-dependent transcriptional cascades. Moreover, our data additionally reveal NPM1 to be a potent drug target for EBV-associated diseases.

## Materials and Methods

### Expression plasmids and cloning

The expression vector of the GST-EBNA2 (GST-E2) acidic domain (AD) was previously described [8]. The remaining GST-E2 expression plasmids were generated by subcloning the indicated EBNA2 flanking sequences, which were amplified by polymerase chain reaction (PCR) using the appropriate corresponding primer pairs, into the *BamH*I and *EcoR*I sites of pGEX-2TK (GE Healthcare). The expression vectors of flag-tagged wild type NPM1 (FNPM1) or its derivatives of truncated deletion mutants were gifts of Dr. Charles J. Sherff (Howard Hughes Medical Institute and Department of Genetics & Tumor Cell Biology, St. Jude Children's Research Hospital). The flag-tagged expression plasmids of the NPM1 point mutants were generated using the QuikChange Site-Directed Mutagenesis Kit (Stratagene) instruction manual. The GFP-NPM1 expression vector was generated by inserting the entire coding sequence of NPM1 into the *Xho*I and *BamH*I sites of eGFP-C1 (TakaraBio USA). The expression vectors of EBNA2 (E2) and EBNALP (LP) and the reporter plasmids LMP1-Luc and CMV-βGal were described previously [10], [45]. The reporter plasmid of NPM1-Luc was generated by subcloning the promoter fragment −910 to +49 into the *Kpn*I and *Xho*I sites of the PGL3 vector (Promega).

### Cell culture, transfection, and cell-based reporter assays

IB4 is an EBV-transformed LCL [46], whereas LCL1-3 are previously established cell lines from the laboratory. AKATA and BJAB are EBV latently infected type I and non-infected Burkitt's Lymphoma (BL) cell lines [47], [48]. Both LCLs and BL cell lines were cultured in RPMI-1640 (Life Technology) supplemented with 10% fetal calf serum (FCS) (R10 medium) (Biological Inc.), while HeLa and 293T cells were maintained in DMEM (Life Technology) supplemented with 10% FCS. For the ATP depletion assay, the culture medium for IB4 or BJAB cells was replaced with glucose (−) RPMI640 (Life Technology) supplemented with 10% FCS, 2 mM deoxyglucose (Sigma), and 300 nM antimycin A (Sigma) and cultured for 2 hours or the indicated time before the assays were completed.

The transfection procedure carried out in this study has been previously described [9], [45]. The EBNA2 and LMP1-Luc reporter co-expressing BJAB stable clones were established by co-transfection of pSG5-EBNA2 (E2), LMP1-Luc, and pIRES-puro (TakaraBio USA) expression plasmids into BJAB cells and selected with 5 ng/ml puromycin. HeLa (2×10^5^) were transfected with 0.5 µg of the GFP-NPM1 and EBNA2 expression vectors, whereas 293T cells (2×10^6^) were transfected with 3 µg FNPM1 or its derivative mutant expression vectors and 3 µg E2 or control empty vector using Lipofectamine 2000 (Life Technology) following the manufacturer's protocol. For the EBNA2-inducing LMP1-Luc activity assay, appropriate amounts of the necessary expression vectors, LMP1-Luc reporter plasmid, and internal control CMV-βGal plasmid were co-transfected into BJAB cells or NPM1 knockdown cells under the indicated circumstances. Luciferase and β-Gal activities were assayed by Orion L (Berthold). The data are represented as the mean ± the standard error of the mean (SEM) from three independent experiments. Whenever necessary, statistical comparisons were performed by one-way ANOVA variance analyses. A p-value of less than 0.05 was considered to be statistically significant.

### Production of the recombinant proteins and the protein binding affinity assay

GST or GST-E2s were expressed in *E. coli* BL21 (DE3) pLys strain (Stratagene) and purified by affinity chromatography analysis using GST-sepharose (GE Healthcare), and each of the purified recombinant proteins was used as the protein bait to pull down proteins from IB4 cell lysates. Furthermore, cell lysates from BJAB cells that had been transfected with 30 µg of flag-tagged empty vector, wild type, or the indicated truncated deletion mutants of NPM1 were further employed for protein affinity pull-down assays using GST and GST-E2 aa 300–360 recombinant proteins. The cellular proteins that were bound to each of the GST-E2s were analyzed by SDS-polyacrylamide gel electrophoresis (SDS-PAGE) followed by LC-MS/MS (Protech Taiwan) or immunoblot analysis.

### Primary B cell isolation and EBV infection

Primary B cells were isolated from peripheral blood mononuclear cells (PBMC) using Dynabeads Untouched (Life Technologies) following the manufacturer's suggested protocol. EBV infectious particles were induced and purified from the B95-8 Z-HT cell line as previously described [49]. Primary B cells (5×10^4^ per 100 µl) were resuspended in RPMI 1640 supplemented with 15% fetal calf serum (FCS), 2 mM L-glutamine, and penicillin/streptomycin and aliquoted into a 96-well plate. One hundred microliters of virus or PBS (mock infection) was used for each infection.

### Immunofluorescence microscopy and flow cytometry

Immunofluorescence (IF) analysis was performed according to the immunostaining protocol described previously [50] using the EBNA2, NPM1, and c-MYC-specific antibodies vC-20 (Santa Cruz), C-19 (Santa Cruz), and α c-MYC (Millipore), respectively. In the co-immunostaining assay, rhodamine-conjugated goat anti-mouse and FITC-conjugated goat anti-rabbit (Kirkegaard & Perry Laboratories, Inc.) antibodies were used as fluorochromes, and DNA was counterstained with DRAQ5 (Bio Status) or DAPI (Sigma).

The nuclear co-localization of EBNA2 and NPM1 was visualized by confocal microscope (LEICA TCS SP2 AOBS). For the virus infection assay, primary B cells infected with EBV or mock (PBS) were immunostained with the appropriate antibodies followed a counterstaining protocol with the corresponding secondary antibodies, which were conjugated with FITC or rhodamine. The immunostained cells were visualized using the Cell Imaging Station (Life Technologies) or analyzed by Guava flow cytometry (Millipore).

### Lentiviral vector production and cell transduction

Lentiviral shRNA expression plasmids of NPM1 were purchased from the National RNAi Core Facility at Academia Sinica, Taiwan. The production of the shRNA-expressing lentiviral vectors was carried out by following the protocol suggested by the manufacturer. To silence NPM1, 5×10^5^ BJAB BL cells or IB4 cells per ml were cultured in six-well culture dishes and transduced with 1 ml of lentivirus supernatant in the presence of 8 µg/mL of polybrene. Each transfectant was replenished with new media 48 hours after transduction and maintained for another 48 hours, followed by selection with 5 ng/ml of puromycin.

### Chromatin immunoprecipitation (ChIP), streptavidin and ATP-agarose mediated pull-down analyses

IB4 cells or EBNA2/LMP1-Luc co-expressing BJAB cells stably transduced with NPM1 shRNAs or scrambled shRNA (1×10^7^) were subjected to an enzymatic shearing protocol (Active motif) suggested by the manufacturer followed by a subsequent chromatin immunoprecipitation (ChIP) analysis using antibodies for NPM1 (5E3; from Abcam), EBNA2 (PE2; from Abcam), or RBP-Jκ (from Genetext). The promoter occupancy of EBNA2, NPM1, and RBP-Jκ was monitored by PCR or quantitative PCR (qPCR) analysis for the corresponding promoter fragments. The primers for this assay are listed in Figure S4C. The ChIP assay kit for GAPDH control was purchased from Millipore. The protocol for qPCR was performed using the Eco Real-Time PCR system (Illumina) as previously described [31]. The promoter occupancy that was identified for each protein or IgG control was normalized by the input DNA and formulated as a percentage of the input DNA. On the other hand, the promoter occupancy of transfected FNPM1 or its mutants were normalized to the IgG control. The LMP1 promoter DNA or control oriP DNA was amplified by PCR using a pair of the corresponding biotin-labeled or non-biotin-labeled primers [31]. Cell lysates from 1×10^7^ or 1×10^8^ IB4 cells were prepared and subjected to a streptavidin (Life Technology) or an ATP-agarose (Sigma) -mediated pull-down assay. The proteins that were bound to each target DNA fragment or ATP-agarose were monitored by immunoblotting with the appropriate antibodies.

### Measurements of cell proliferation

Normal IB4 LCL and NPM1 knockdown IB4 cells (5×10^4^ per 200 µl) or BJAB cells and NPM1 knockdown BJAB cells (10^4^ per 200 µl) were aliquoted in triplicate into 96-well plates. Viable cells were counted using the trypan blue exclusion method every 24 hours for five consecutive days.

## Supporting Information

Figure S1 **The protein-protein interaction map of EBNA2, as it relates to** **Figure 1** **.** A). Cellular proteins from IB4 cell lysates pulled down by GST or GST-E2s were subjected to 4–20% gradient SDS-PAGE gel and visualized with Coomassie blue staining. Specific bands are marked with individual arrowheads. B). The proteins contained in each unique gel band were identified by LC-MS/MS analysis. The description of the proteins identified in each gel band and the coverage of the peptide sequences for the corresponding proteins are shown.
(PPT)

Figure S2 **Expression of NPM1 and c-MYC is induced in EBV-infected B cells, in relation to** **Figure 3** **.** A). Primary B cells (5×10^4^) were infected with EBV or PBS (mock) and subjected to an IF staining protocol for monitoring the expression of EBNA2 (Green) and NPM1 (Red) at 3 or 7 dai. Nuclei were counterstained with DAPI. The immunostained cells were visualized with fluorescence microscopy and quantified by flow cytometry (See table S1). B). The same protocol that was described in (A) was carried out to identify the expression of EBNA2 (Green) and its target gene c-MYC (Red). Both color and grayscale images are shown. C). Co-immunostaining of the newly established LCL (LCL-New) or primary B cells using antibodies for EBNA2 and NPM1. The immunostained cells were visualized by confocal microscopy.
(PPT)

Figure S3 **The EBNA2 binding domain of NPM1 associated with RBPJ, in relation to** **Figure 5** **.** BJAB cells transfected with the expression vector of FNPM1 or its truncated deletion mutant derivatives were subjected to M2-sepharose-mediated IP analysis followed by immunoblotting with antibodies for the flag epitope (M2) and RBPJ. Two percent input of endogenous RBPJ is shown.
(PPT)

Figure S4 **Dissociation of EBNA2 and NPM1 from the cognate response elements by ATP-depletion, in relation to** **Figure 6** **.** A). The accumulation of EBNA2, NPM1, RBPJ, and control IgG at the LMP1 promoter in IB4 cells treated with or without ATP-depletion was identified by ChIP-qPCR assay. Relative promoter occupancy of each protein is expressed as a percentage of the input DNA. B). The same protocol that was described in (A) was used to identify the abundance of H3ac at the GAPDH promoter. Error bars represent the standard deviation of triplicate samples for this and subsequent ChIP assays. C). The list of primers used for qPCR assay in this study. D). Transfection-mediated SV40-Luc reporter assay was performed using BJAB cells. Treatment of ATP-depletion was performed at 0.5, 1, 1.5, 2 hours before the luciferase activity assay. The activity of the transfected SV40-Luc reporter plasmid was normalized by the β-gal activity produced by the internal control CMV-β-gal. E). 293T cells that were transfected with each of the expression vectors of GFP, FNPM1, and EBNA2 binding mutants L_102_A, G_105_A, and S_106_A were subjected to ATP-agarose-mediated pull-down assay, respectively. Five percent input of each protein is shown.
(PPT)

Figure S5 **NPM1 binds to EBNA2 and recruits it to the EBV latency-specific LMP1 promoter, in relation to** **Figure 7** **.** BJAB-LMP1-Luc cells were co-transfected with the expression plasmids of EBNA2 and FNPM1 or each EBNA2 binding mutants G_105_A, S_106_A, K_257/263_R were subjected to M2-ChIP assay. The accumulation of each protein at the LMP1 promoter is expressed in relation to its background level of IgG.
(PPT)

Table S1 **The EBV infection assay.** Primary B cells (5×10^4^) were infected with EBV or PBS (Mock) and subjected to an IF staining protocol using antibodies for EBNA2 (v-C20), NPM1 (C-19), or c-MYC at 0, 3 or 7 days after infection (dai) followed by a donkey anti-goat antibody conjugated to FITC (Green) or a goat anti-mouse antibody conjugated to rhodamine (red). Nuclei were counterstained with DAPI. The immunostained cells were quantified by flow cytometry. The summary data from immunostaining assays are shown.
(PPT)

## References

[1] Kieff EaR, A. (2007) Epstein-Barr Virus and Its Replication In: Knipe DaHP, editor. Fields Virology. 5th edition. Philadelphia: Lippincott, Williams, and WIlkins. pp. 2603–2700.

[2] Laux G, Adam B, Strobl LJ, Moreau-Gachelin F (1994) The Spi-1/PU.1 and Spi-B ets family transcription factors and the recombination signal binding protein RBP-J kappa interact with an Epstein-Barr virus nuclear antigen 2 responsive cis-element. Embo J 13: 5624–5632. 7988559 10.1002/j.1460-2075.1994.tb06900.x PMC395527

[3] Hsieh JJ, Hayward SD (1995) Masking of the CBF1/RBPJ kappa transcriptional repression domain by Epstein-Barr virus EBNA2. Science 268: 560–563. 7725102 10.1126/science.7725102

[4] Ling PD, Hayward SD (1995) Contribution of conserved amino acids in mediating the interaction between EBNA2 and CBF1/RBPJk. J Virol 69: 1944–1950. 7853539 10.1128/jvi.69.3.1944-1950.1995 PMC188813

[5] Fuentes-Panana EM, Peng R, Brewer G, Tan J, Ling PD (2000) Regulation of the Epstein-Barr virus C promoter by AUF1 and the cyclic AMP/protein kinase A signaling pathway. J Virol 74: 8166–8175. 10933728 10.1128/jvi.74.17.8166-8175.2000 PMC112351

[6] Tong X, Drapkin R, Yalamanchili R, Mosialos G, Kieff E (1995) The Epstein-Barr virus nuclear protein 2 acidic domain forms a complex with a novel cellular coactivator that can interact with TFIIE. Mol Cell Biol 15: 4735–4744. 7651391 10.1128/mcb.15.9.4735 PMC230717

[7] Tong X, Drapkin R, Reinberg D, Kieff E (1995) The 62- and 80-kDa subunits of transcription factor IIH mediate the interaction with Epstein-Barr virus nuclear protein 2. Proc Natl Acad Sci U S A 92: 3259–3263. 7724549 10.1073/pnas.92.8.3259 PMC42145

[8] Tong X, Wang F, Thut CJ, Kieff E (1995) The Epstein-Barr virus nuclear protein 2 acidic domain can interact with TFIIB, TAF40, and RPA70 but not with TATA-binding protein. J Virol 69: 585–588. 7983760 10.1128/jvi.69.1.585-588.1995 PMC188615

[9] Peng CW, Xue Y, Zhao B, Johannsen E, Kieff E, et al (2004) Direct interactions between Epstein-Barr virus leader protein LP and the EBNA2 acidic domain underlie coordinate transcriptional regulation. Proc Natl Acad Sci U S A 101: 1033–1038. 14732686 10.1073/pnas.0307808100 PMC327146

[10] Peng CW, Zhao B, Kieff E (2004) Four EBNA2 domains are important for EBNALP coactivation. J Virol 78: 11439–11442. 15452270 10.1128/JVI.78.20.11439-11442.2004 PMC521825

[11] Gordadze AV, Onunwor CW, Peng R, Poston D, Kremmer E, et al (2004) EBNA2 amino acids 3 to 30 are required for induction of LMP-1 and immortalization maintenance. J Virol 78: 3919–3929. 15047808 10.1128/JVI.78.8.3919-3929.2004 PMC374290

[12] Wang L, Grossman SR, Kieff E (2000) Epstein-Barr virus nuclear protein 2 interacts with p300, CBP, and PCAF histone acetyltransferases in activation of the LMP1 promoter. Proc Natl Acad Sci U S A 97: 430–435. 10618435 10.1073/pnas.97.1.430 PMC26680

[13] Zhou S, Fujimuro M, Hsieh JJ, Chen L, Hayward SD (2000) A role for SKIP in EBNA2 activation of CBF1-repressed promoters. J Virol 74: 1939–1947. 10644367 10.1128/jvi.74.4.1939-1947.2000 PMC111672

[14] Barth S, Liss M, Voss MD, Dobner T, Fischer U, et al (2003) Epstein-Barr virus nuclear antigen 2 binds via its methylated arginine-glycine repeat to the survival motor neuron protein. J Virol 77: 5008–5013. 12663808 10.1128/JVI.77.8.5008-5013.2003 PMC152127

[15] Callebaut I, Mornon JP (1997) The human EBNA-2 coactivator p100: multidomain organization and relationship to the staphylococcal nuclease fold and to the tudor protein involved in Drosophila melanogaster development. Biochem J 321 (Pt 1) 125–132. 9003410 10.1042/bj3210125 PMC1218045

[16] Voss MD, Hille A, Barth S, Spurk A, Hennrich F, et al (2001) Functional cooperation of Epstein-Barr virus nuclear antigen 2 and the survival motor neuron protein in transactivation of the viral LMP1 promoter. J Virol 75: 11781–11790. 11689659 10.1128/JVI.75.23.11781-11790.2001 PMC114764

[17] Wu DY, Krumm A, Schubach WH (2000) Promoter-specific targeting of human SWI-SNF complex by Epstein-Barr virus nuclear protein 2. J Virol 74: 8893–8903. 10982332 10.1128/jvi.74.19.8893-8903.2000 PMC102084

[18] Liu X, Liu Z, Jang SW, Ma Z, Shinmura K, et al (2007) Sumoylation of nucleophosmin/B23 regulates its subcellular localization, mediating cell proliferation and survival. Proc Natl Acad Sci U S A 104: 9679–9684. 17535915 10.1073/pnas.0701806104 PMC1887583

[19] Feuerstein N, Chan PK, Mond JJ (1988) Identification of numatrin, the nuclear matrix protein associated with induction of mitogenesis, as the nucleolar protein B23. Implication for the role of the nucleolus in early transduction of mitogenic signals. J Biol Chem 263: 10608–10612. 3392030

[20] Li Z, Boone D, Hann SR (2008) Nucleophosmin interacts directly with c-Myc and controls c-Myc-induced hyperproliferation and transformation. Proc Natl Acad Sci U S A 105: 18794–18799. 19033198 10.1073/pnas.0806879105 PMC2596228

[21] Grisendi S, Mecucci C, Falini B, Pandolfi PP (2006) Nucleophosmin and cancer. Nat Rev Cancer 6: 493–505. 16794633 10.1038/nrc1885

[22] Swaminathan V, Kishore AH, Febitha KK, Kundu TK (2005) Human histone chaperone nucleophosmin enhances acetylation-dependent chromatin transcription. Mol Cell Biol 25: 7534–7545. 16107701 10.1128/MCB.25.17.7534-7545.2005 PMC1190275

[23] Enomoto T, Lindstrom MS, Jin A, Ke H, Zhang Y (2006) Essential role of the B23/NPM core domain in regulating ARF binding and B23 stability. J Biol Chem 281: 18463–18472. 16679321 10.1074/jbc.M602788200

[24] Koike A, Nishikawa H, Wu W, Okada Y, Venkitaraman AR, et al (2010) Recruitment of phosphorylated NPM1 to sites of DNA damage through RNF8-dependent ubiquitin conjugates. Cancer Res 70: 6746–6756. 20713529 10.1158/0008-5472.CAN-10-0382

[25] Okuwaki M, Matsumoto K, Tsujimoto M, Nagata K (2001) Function of nucleophosmin/B23, a nucleolar acidic protein, as a histone chaperone. FEBS Lett 506: 272–276. 11602260 10.1016/s0014-5793(01)02939-8

[26] Korgaonkar C, Hagen J, Tompkins V, Frazier AA, Allamargot C, et al (2005) Nucleophosmin (B23) targets ARF to nucleoli and inhibits its function. Mol Cell Biol 25: 1258–1271. 15684379 10.1128/MCB.25.4.1258-1271.2005 PMC548001

[27] Kurki S, Peltonen K, Latonen L, Kiviharju TM, Ojala PM, et al (2004) Nucleolar protein NPM interacts with HDM2 and protects tumor suppressor protein p53 from HDM2-mediated degradation. Cancer Cell 5: 465–475. 15144954 10.1016/s1535-6108(04)00110-2

[28] Di Fiore PP (2008) Playing both sides: nucleophosmin between tumor suppression and oncogenesis. J Cell Biol 182: 7–9. 18625839 10.1083/jcb.200806069 PMC2447893

[29] Leotoing L, Meunier L, Manin M, Mauduit C, Decaussin M, et al (2008) Influence of nucleophosmin/B23 on DNA binding and transcriptional activity of the androgen receptor in prostate cancer cell. Oncogene 27: 2858–2867. 18037965 10.1038/sj.onc.1210942

[30] Bertwistle D, Sherr CJ (2007) Regulation of the Arf tumor suppressor in Emicro-Myc transgenic mice: longitudinal study of Myc-induced lymphomagenesis. Blood 109: 792–794. 16968893 10.1182/blood-2006-07-033985

[31] Chen YL, Tsai HL, Peng CW (2012) EGCG debilitates the persistence of EBV latency by reducing the DNA binding potency of nuclear antigen 1. Biochem Biophys Res Commun 417: 1093–1099. 22226960 10.1016/j.bbrc.2011.12.104

[32] Choi JW, Lee SB, Kim CK, Lee KH, Cho SW, et al (2008) Lysine 263 residue of NPM/B23 is essential for regulating ATP binding and B23 stability. FEBS Lett 582: 1073–1080. 18319061 10.1016/j.febslet.2008.02.059

[33] Chang JH, Lin JY, Wu MH, Yung BY (1998) Evidence for the ability of nucleophosmin/B23 to bind ATP. Biochem J 329 (Pt 3) 539–544. 9445380 10.1042/bj3290539 PMC1219074

[34] Lindstrom MS (2011) NPM1/B23: A Multifunctional Chaperone in Ribosome Biogenesis and Chromatin Remodeling. Biochem Res Int 2011: 195209. 21152184 10.1155/2011/195209 PMC2989734

[35] Feuerstein N, Mond JJ (1987) “Numatrin,” a nuclear matrix protein associated with induction of proliferation in B lymphocytes. J Biol Chem 262: 11389–11397. 3301855

[36] Zeller KI, Haggerty TJ, Barrett JF, Guo Q, Wonsey DR, et al (2001) Characterization of nucleophosmin (B23) as a Myc target by scanning chromatin immunoprecipitation. J Biol Chem 276: 48285–48291. 11604407 10.1074/jbc.M108506200

[37] Faumont N, Durand-Panteix S, Schlee M, Gromminger S, Schuhmacher M, et al (2009) c-Myc and Rel/NF-kappaB are the two master transcriptional systems activated in the latency III program of Epstein-Barr virus-immortalized B cells. J Virol 83: 5014–5027. 19264782 10.1128/JVI.02264-08 PMC2682111

[38] Kaiser C, Laux G, Eick D, Jochner N, Bornkamm GW, et al (1999) The proto-oncogene c-myc is a direct target gene of Epstein-Barr virus nuclear antigen 2. J Virol 73: 4481–4484. 10196351 10.1128/jvi.73.5.4481-4484.1999 PMC104340

[39] Huang WH, Yung BY, Syu WJ, Lee YH (2001) The nucleolar phosphoprotein B23 interacts with hepatitis delta antigens and modulates the hepatitis delta virus RNA replication. J Biol Chem 276: 25166–25175. 11309377 10.1074/jbc.M010087200

[40] Tsuda Y, Mori Y, Abe T, Yamashita T, Okamoto T, et al (2006) Nucleolar protein B23 interacts with Japanese encephalitis virus core protein and participates in viral replication. Microbiol Immunol 50: 225–234. 16547420 10.1111/j.1348-0421.2006.tb03789.x

[41] Okuwaki M, Iwamatsu A, Tsujimoto M, Nagata K (2001) Identification of nucleophosmin/B23, an acidic nucleolar protein, as a stimulatory factor for in vitro replication of adenovirus DNA complexed with viral basic core proteins. J Mol Biol 311: 41–55. 11469856 10.1006/jmbi.2001.4812

[42] Sarek G, Jarviluoma A, Moore HM, Tojkander S, Vartia S, et al (2010) Nucleophosmin phosphorylation by v-cyclin-CDK6 controls KSHV latency. PLoS Pathog 6: e1000818. 20333249 10.1371/journal.ppat.1000818 PMC2841626

[43] Gadad SS, Rajan RE, Senapati P, Chatterjee S, Shandilya J, et al (2011) HIV-1 infection induces acetylation of NPM1 that facilitates Tat localization and enhances viral transactivation. J Mol Biol 410: 997–1007. 21763502 10.1016/j.jmb.2011.04.009

[44] Zimber-Strobl U, Strobl LJ (2001) EBNA2 and Notch signalling in Epstein-Barr virus mediated immortalization of B lymphocytes. Semin Cancer Biol 11: 423–434. 11669604 10.1006/scbi.2001.0409

[45] Peng CW, Zhao B, Chen HC, Chou ML, Lai CY, et al (2007) Hsp72 up-regulates Epstein-Barr virus EBNALP coactivation with EBNA2. Blood 109: 5447–5454. 17341665 10.1182/blood-2006-08-040634 PMC1890828

[46] King W, Thomas-Powell AL, Raab-Traub N, Hawke M, Kieff E (1980) Epstein-Barr virus RNA. V. Viral RNA in a restringently infected, growth-transformed cell line. J Virol 36: 506–518. 6253674 10.1128/jvi.36.2.506-518.1980 PMC353668

[47] Menezes J, Leibold W, Klein G, Clements G (1975) Establishment and characterization of an Epstein-Barr virus (EBC)- negative lymphoblastoid B cell line (BJA-B) from an exceptional, EBV- genome-negative African Burkitt's lymphoma. Biomedicine 22: 276–284. 179629

[48] Takada K, Horinouchi K, Ono Y, Aya T, Osato T, et al (1991) An Epstein-Barr virus-producer line Akata: establishment of the cell line and analysis of viral DNA. Virus Genes 5: 147–156. 1647567 10.1007/BF00571929

[49] Johannsen E, Luftig M, Chase MR, Weicksel S, Cahir-McFarland E, et al (2004) Proteins of purified Epstein-Barr virus. Proc Natl Acad Sci U S A 101: 16286–16291. 15534216 10.1073/pnas.0407320101 PMC528973

[50] Cooper A, Johannsen E, Maruo S, Cahir-McFarland E, Illanes D, et al (2003) EBNA3A association with RBP-Jkappa down-regulates c-myc and Epstein-Barr virus-transformed lymphoblast growth. J Virol 77: 999–1010. 12502816 10.1128/JVI.77.2.999-1010.2003 PMC140836

