# Supplementary material for: The Nuclear Chaperone Nucleophosmin Escorts an Epstein-Barr Virus Nuclear Antigen to Establish Transcriptional Cascades for Latent Infection in Human B Cells
Source: PLoS Pathog. 2012 Dec 13;8(12):e1003084. doi: 10.1371/journal.ppat.1003084 (PMC3521654; doi:10.1371/journal.ppat.1003084)
Supplement: Figure S1 — The protein-protein interaction map of EBNA2, as it relates to Figure 1 . A). Cellular proteins from IB4 cell lysates pulled down by GST or GST-E2s were subjected to 4–20% gradient SDS-PAGE gel and visualized with Coomassie blue staining. Specific bands are marked with individual arrowheads. B). The proteins contained in each unique gel band were identified by LC-MS/MS analysis. The description of the proteins identified in each gel band and the coverage of the peptide sequences for the corresponding proteins are shown. (PPT) [file ppat.1003084.s001.ppt]

## Slide 1
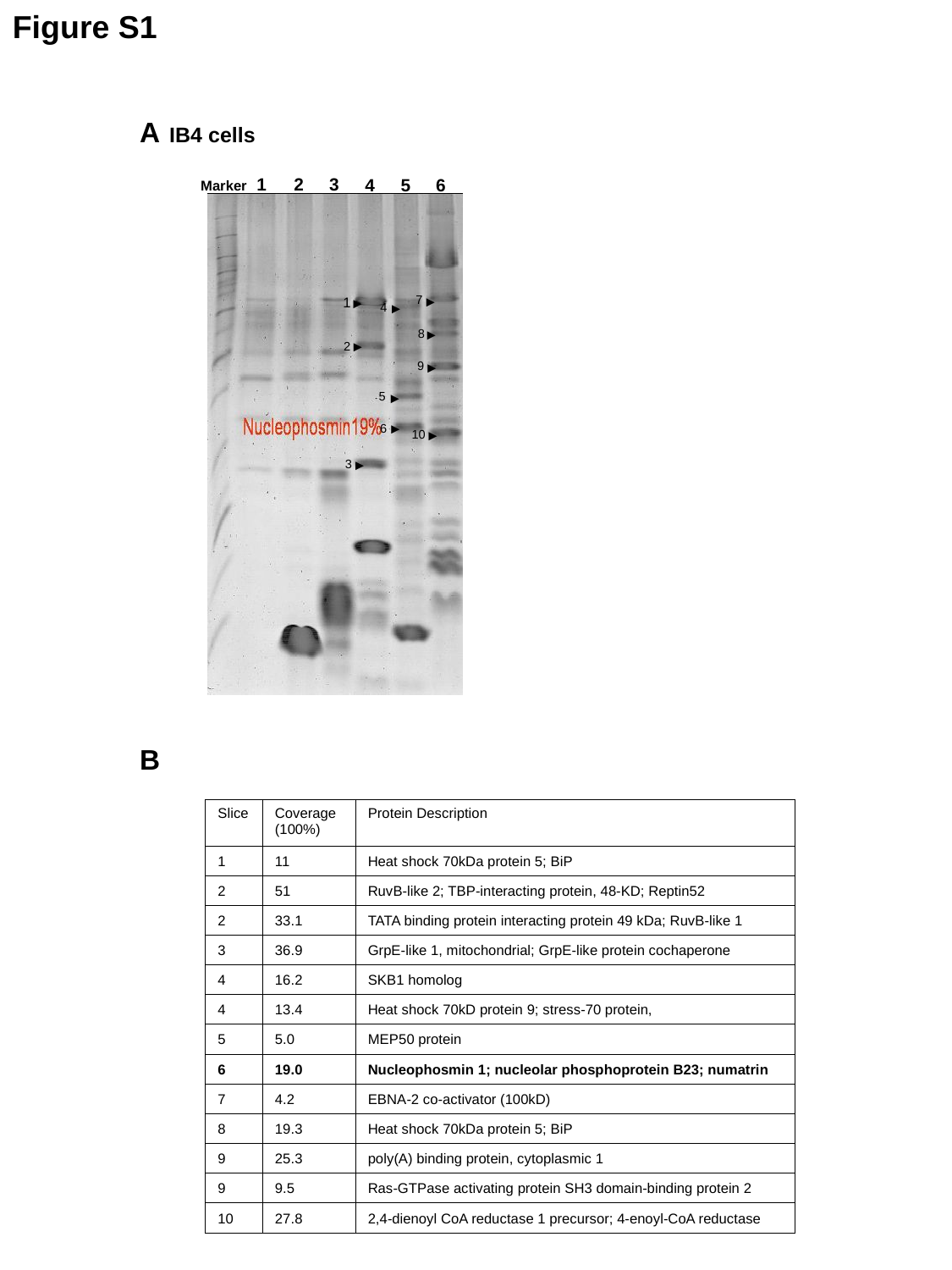

Figure S1
A
 IB4 cells
1
2
3
4
5
6
Marker
7
►
1
►
4
►
►
8
►
2
9
►
5
►
►
6
►
10
3
►
B
| Slice | Coverage (100%) | Protein Description |
| --- | --- | --- |
| 1 | 11 | Heat shock 70kDa protein 5; BiP |
| 2 | 51 | RuvB-like 2; TBP-interacting protein, 48-KD; Reptin52 |
| 2 | 33.1 | TATA binding protein interacting protein 49 kDa; RuvB-like 1 |
| 3 | 36.9 | GrpE-like 1, mitochondrial; GrpE-like protein cochaperone |
| 4 | 16.2 | SKB1 homolog |
| 4 | 13.4 | Heat shock 70kD protein 9; stress-70 protein, |
| 5 | 5.0 | MEP50 protein |
| 6 | 19.0 | Nucleophosmin 1; nucleolar phosphoprotein B23; numatrin |
| 7 | 4.2 | EBNA-2 co-activator (100kD) |
| 8 | 19.3 | Heat shock 70kDa protein 5; BiP |
| 9 | 25.3 | poly(A) binding protein, cytoplasmic 1 |
| 9 | 9.5 | Ras-GTPase activating protein SH3 domain-binding protein 2 |
| 10 | 27.8 | 2,4-dienoyl CoA reductase 1 precursor; 4-enoyl-CoA reductase |
