# Supplementary material for: The Nuclear Chaperone Nucleophosmin Escorts an Epstein-Barr Virus Nuclear Antigen to Establish Transcriptional Cascades for Latent Infection in Human B Cells
Source: PLoS Pathog. 2012 Dec 13;8(12):e1003084. doi: 10.1371/journal.ppat.1003084 (PMC3521654; doi:10.1371/journal.ppat.1003084)
Supplement: Figure S2 — Expression of NPM1 and c-MYC is induced in EBV-infected B cells, in relation to Figure 3 . A). Primary B cells (5×10^4^) were infected with EBV or PBS (mock) and subjected to an IF staining protocol for monitoring the expression of EBNA2 (Green) and NPM1 (Red) at 3 or 7 dai. Nuclei were counterstained with DAPI. The immunostained cells were visualized with fluorescence microscopy and quantified by flow cytometry (See table S1). B). The same protocol that was described in (A) was carried out to identify the expression of EBNA2 (Green) and its target gene c-MYC (Red). Both color and grayscale images are shown. C). Co-immunostaining of the newly established LCL (LCL-New) or primary B cells using antibodies for EBNA2 and NPM1. The immunostained cells were visualized by confocal microscopy. (PPT) [file ppat.1003084.s002.ppt]

## Slide 1
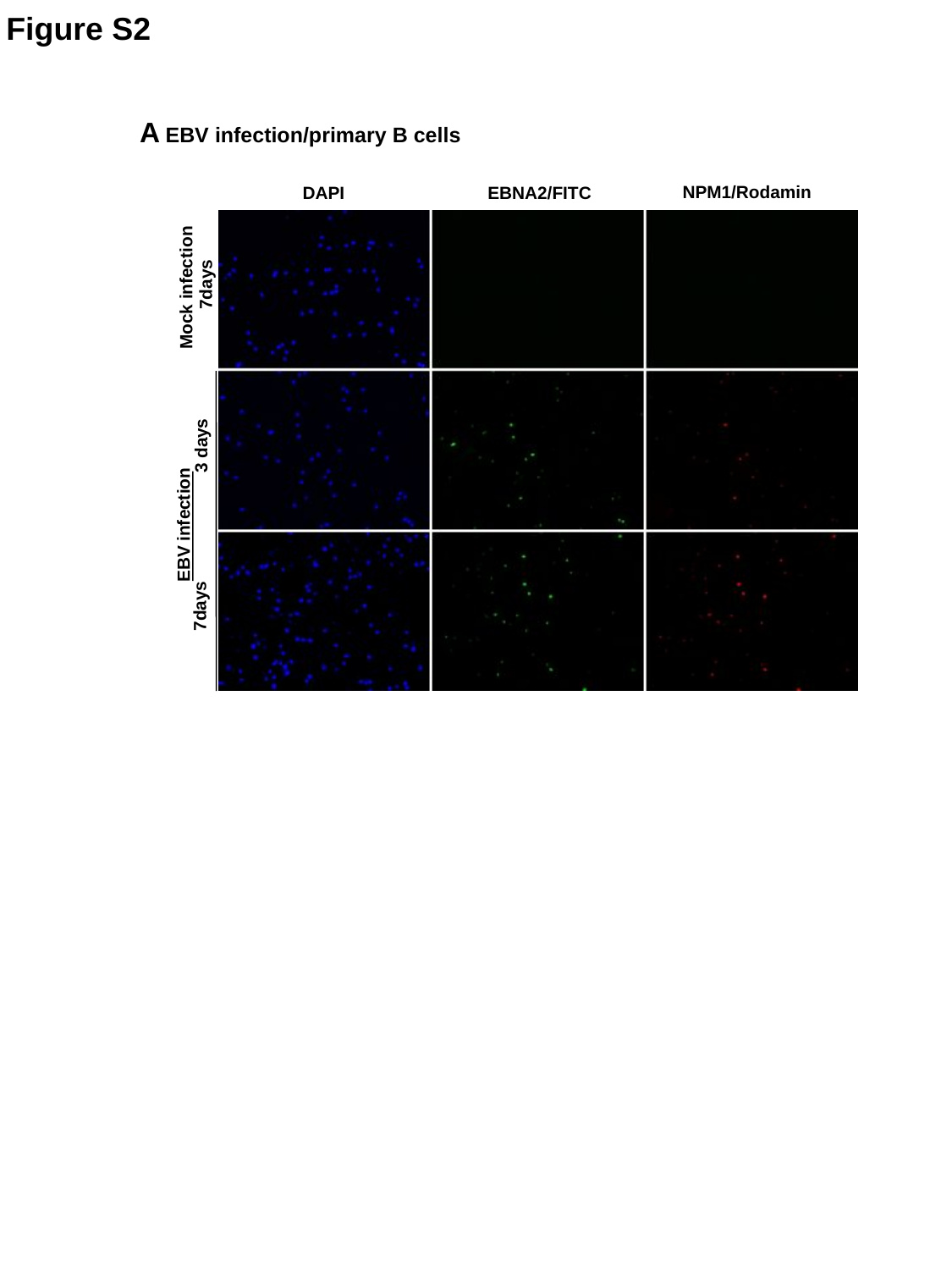

Figure S2
A
EBV infection/primary B cells
NPM1/Rodamin
DAPI
EBNA2/FITC
7days
Mock infection
3 days
EBV infection
7days

## Slide 2
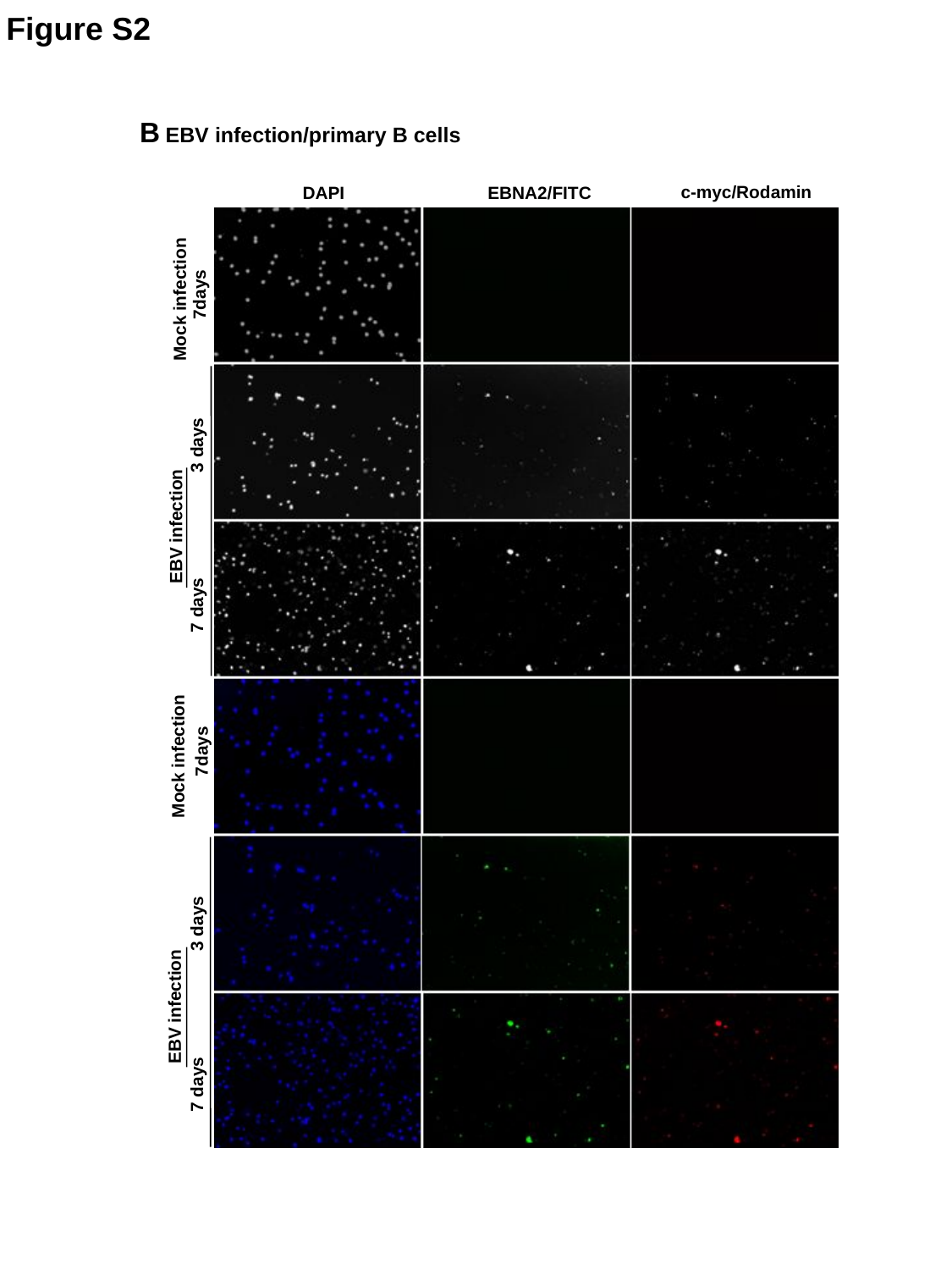

Figure S2
B
EBV infection/primary B cells
c-myc/Rodamin
DAPI
EBNA2/FITC
7days
Mock infection
3 days
EBV infection
7 days
7days
Mock infection
3 days
EBV infection
7 days

## Slide 3
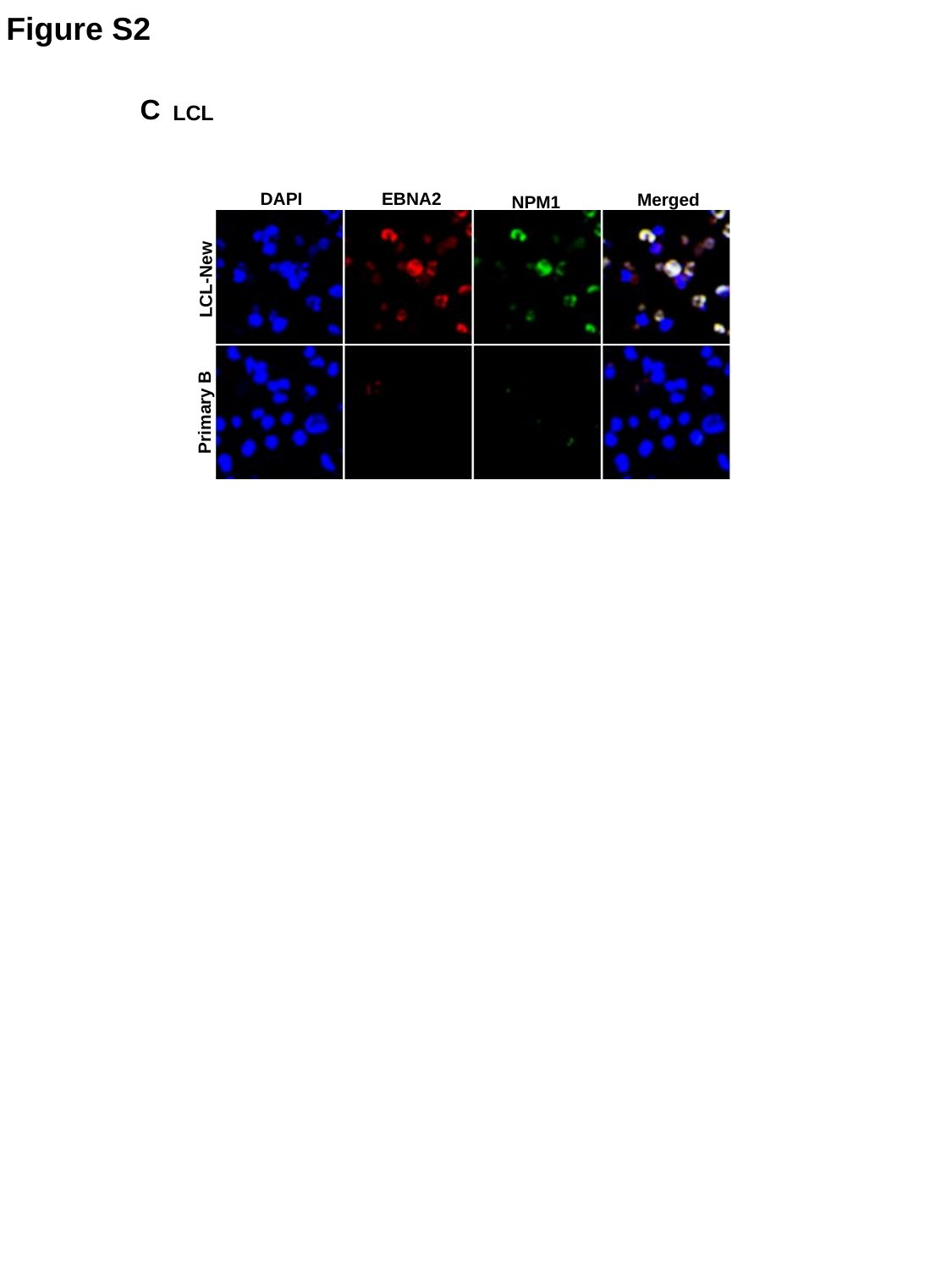

Figure S2
C
LCL
DAPI
EBNA2
Merged
NPM1
LCL-New
Primary B
