# Supplementary figures and images for: The Nuclear Chaperone Nucleophosmin Escorts an Epstein-Barr Virus Nuclear Antigen to Establish Transcriptional Cascades for Latent Infection in Human B Cells
Source: PLoS Pathog. 2012 Dec 13;8(12):e1003084. doi: 10.1371/journal.ppat.1003084 (PMC3521654; doi:10.1371/journal.ppat.1003084)

## Slide 1
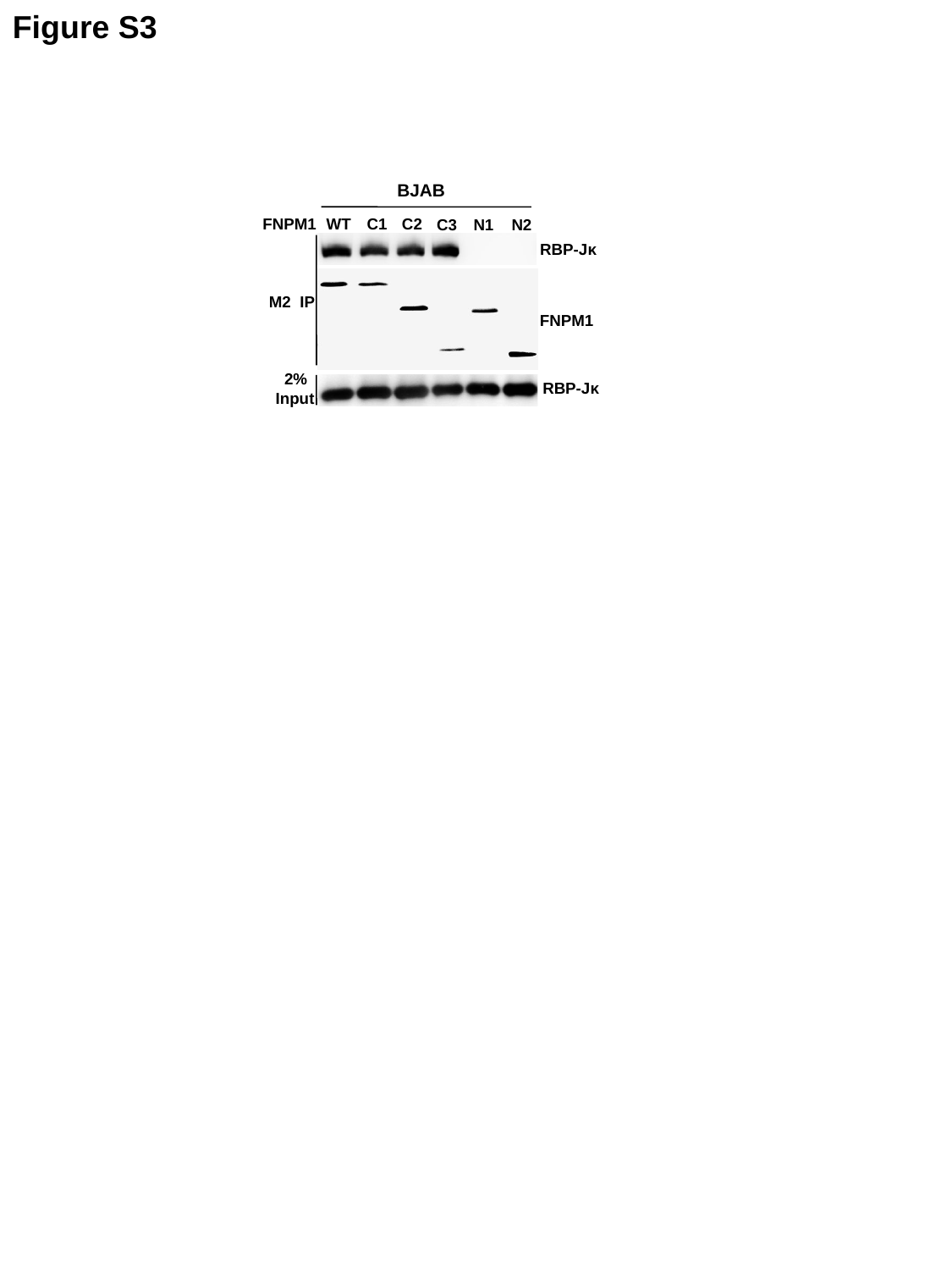

Figure S3
 BJAB
WT
C1
C2
FNPM1
N1
C3
N2
RBP-Jκ
M2 IP
FNPM1
 2% Input
RBP-Jκ

Supplement: Figure S3 — The EBNA2 binding domain of NPM1 associated with RBPJ, in relation to Figure 5 . BJAB cells transfected with the expression vector of FNPM1 or its truncated deletion mutant derivatives were subjected to M2-sepharose-mediated IP analysis followed by immunoblotting with antibodies for the flag epitope (M2) and RBPJ. Two percent input of endogenous RBPJ is shown. (PPT) [file ppat.1003084.s003.ppt]

## Slide 1
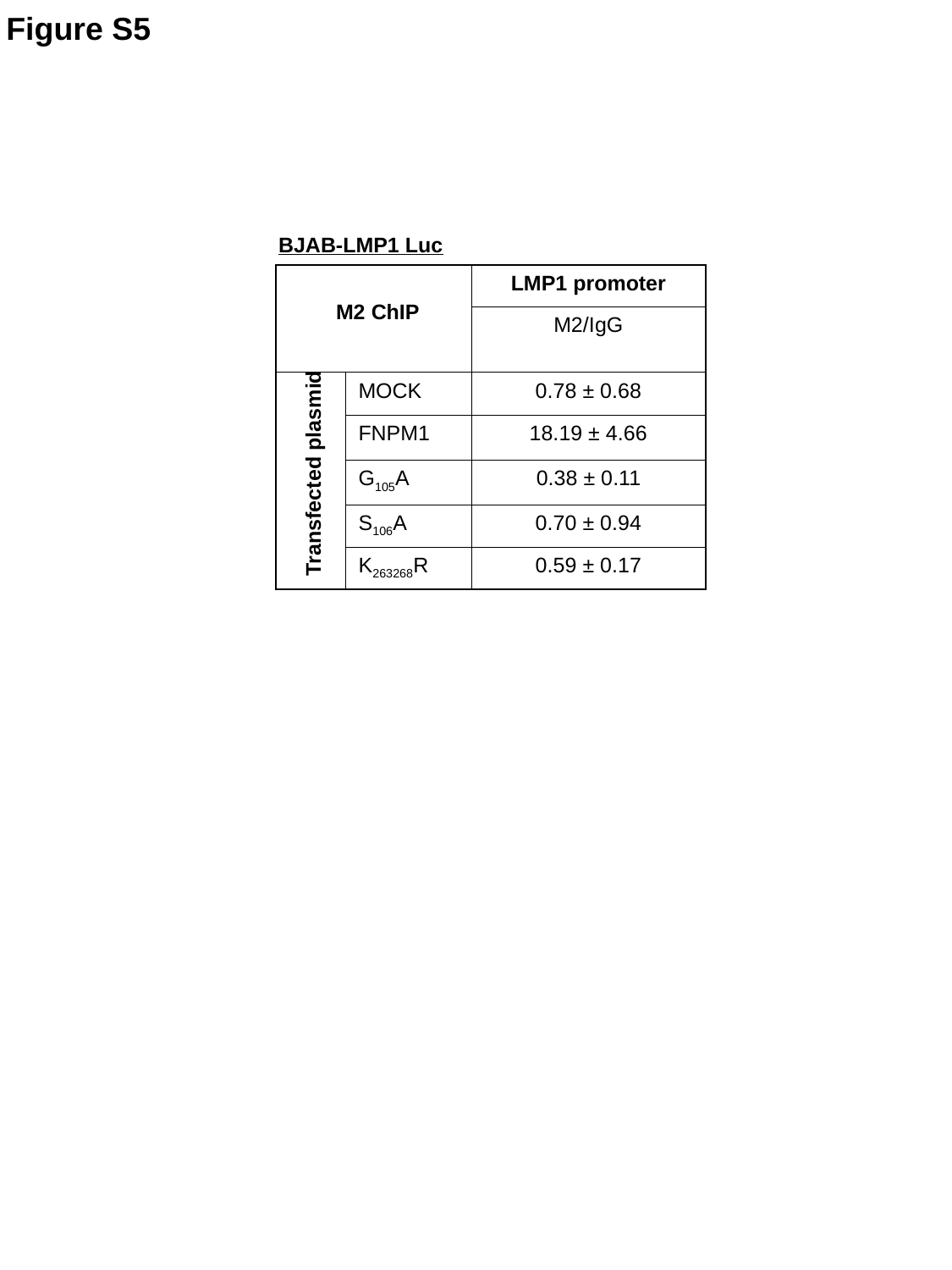

Figure S5
BJAB-LMP1 Luc
| M2 ChIP | | LMP1 promoter |
| --- | --- | --- |
| | | M2/IgG |
| | MOCK | 0.78 ± 0.68 |
| | FNPM1 | 18.19 ± 4.66 |
| | G105A | 0.38 ± 0.11 |
| | S106A | 0.70 ± 0.94 |
| | K263268R | 0.59 ± 0.17 |
Transfected plasmid

Supplement: Figure S5 — NPM1 binds to EBNA2 and recruits it to the EBV latency-specific LMP1 promoter, in relation to Figure 7 . BJAB-LMP1-Luc cells were co-transfected with the expression plasmids of EBNA2 and FNPM1 or each EBNA2 binding mutants G105A, S106A, K257/263R were subjected to M2-ChIP assay. The accumulation of each protein at the LMP1 promoter is expressed in relation to its background level of IgG. (PPT) [file ppat.1003084.s005.ppt]
