# Supplementary material for: The Nuclear Chaperone Nucleophosmin Escorts an Epstein-Barr Virus Nuclear Antigen to Establish Transcriptional Cascades for Latent Infection in Human B Cells
Source: PLoS Pathog. 2012 Dec 13;8(12):e1003084. doi: 10.1371/journal.ppat.1003084 (PMC3521654; doi:10.1371/journal.ppat.1003084)
Supplement: Figure S4 — Dissociation of EBNA2 and NPM1 from the cognate response elements by ATP-depletion, in relation to Figure 6 . A). The accumulation of EBNA2, NPM1, RBPJ, and control IgG at the LMP1 promoter in IB4 cells treated with or without ATP-depletion was identified by ChIP-qPCR assay. Relative promoter occupancy of each protein is expressed as a percentage of the input DNA. B). The same protocol that was described in (A) was used to identify the abundance of H3ac at the GAPDH promoter. Error bars represent the standard deviation of triplicate samples for this and subsequent ChIP assays. C). The list of primers used for qPCR assay in this study. D). Transfection-mediated SV40-Luc reporter assay was performed using BJAB cells. Treatment of ATP-depletion was performed at 0.5, 1, 1.5, 2 hours before the luciferase activity assay. The activity of the transfected SV40-Luc reporter plasmid was normalized by the β-gal activity produced by the internal control CMV-β-gal. E). 293T cells that were transfected with each of the expression vectors of GFP, FNPM1, and EBNA2 binding mutants L102A, G105A, and S106A were subjected to ATP-agarose-mediated pull-down assay, respectively. Five percent input of each protein is shown. (PPT) [file ppat.1003084.s004.ppt]

## Slide 1
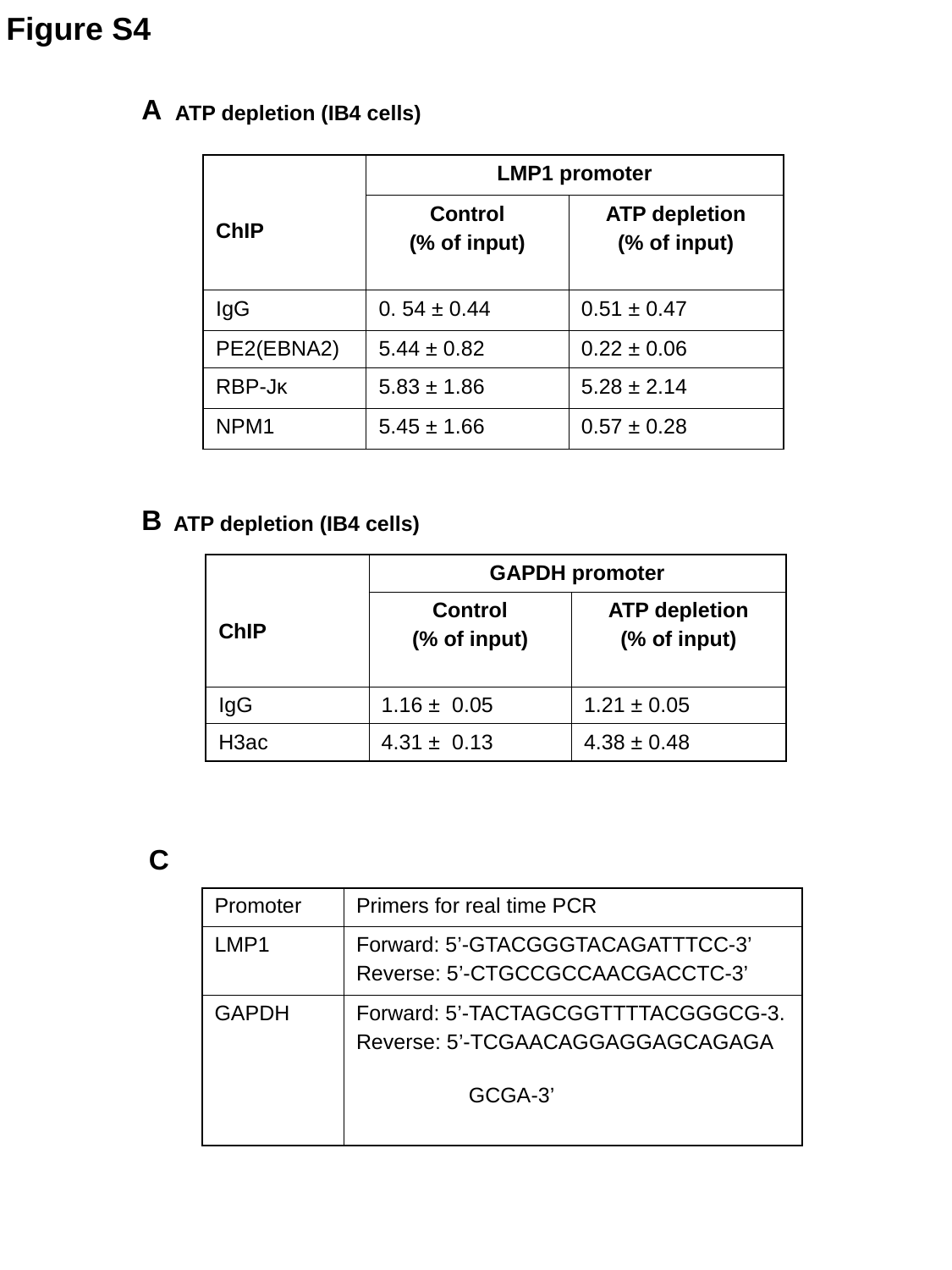

Figure S4
A
ATP depletion (IB4 cells)
| ChIP | LMP1 promoter | |
| --- | --- | --- |
| | Control (% of input) | ATP depletion (% of input) |
| IgG | 0. 54 ± 0.44 | 0.51 ± 0.47 |
| PE2(EBNA2) | 5.44 ± 0.82 | 0.22 ± 0.06 |
| RBP-Jκ | 5.83 ± 1.86 | 5.28 ± 2.14 |
| NPM1 | 5.45 ± 1.66 | 0.57 ± 0.28 |
B
ATP depletion (IB4 cells)
| ChIP | GAPDH promoter | |
| --- | --- | --- |
| | Control (% of input) | ATP depletion (% of input) |
| IgG | 1.16 ± 0.05 | 1.21 ± 0.05 |
| H3ac | 4.31 ± 0.13 | 4.38 ± 0.48 |
C
| Promoter | Primers for real time PCR |
| --- | --- |
| LMP1 | Forward: 5’-GTACGGGTACAGATTTCC-3’ Reverse: 5’-CTGCCGCCAACGACCTC-3’ |
| GAPDH | Forward: 5’-TACTAGCGGTTTTACGGGCG-3. Reverse: 5’-TCGAACAGGAGGAGCAGAGA GCGA-3’ |

## Slide 2
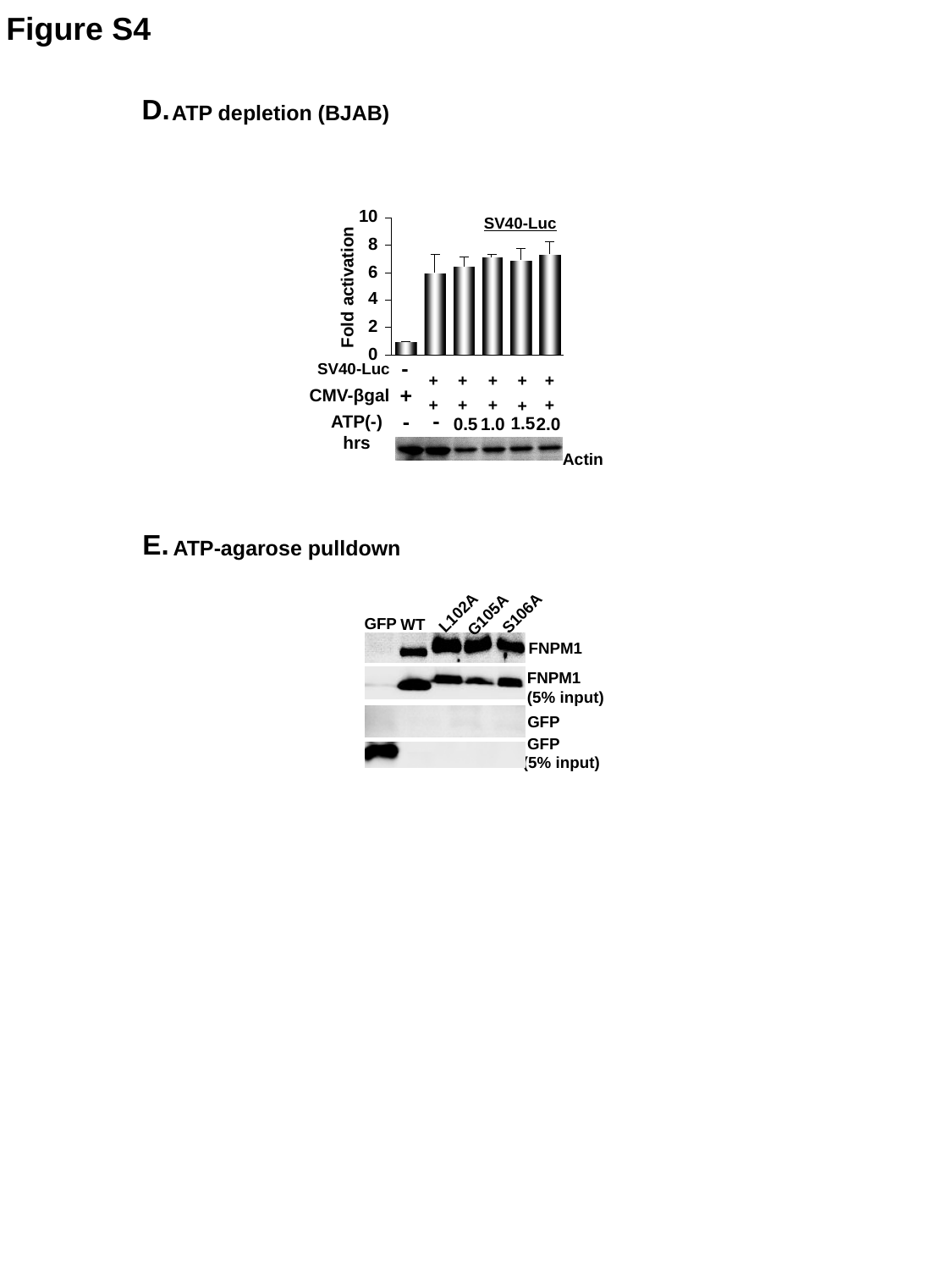

Figure S4
D.
ATP depletion (BJAB)
SV40-Luc
+
+
+
+
+
-
SV40-Luc
+
+
+
+
+
+
CMV-βgal
-
-
ATP(-)
hrs
1.5
0.5
1.0
2.0
Actin
E.
ATP-agarose pulldown
L102A
S106A
G105A
GFP
WT
 FNPM1
 FNPM1
 (5% input)
 GFP
 GFP
(5% input)
