# Supplementary material for: The Nuclear Chaperone Nucleophosmin Escorts an Epstein-Barr Virus Nuclear Antigen to Establish Transcriptional Cascades for Latent Infection in Human B Cells
Source: PLoS Pathog. 2012 Dec 13;8(12):e1003084. doi: 10.1371/journal.ppat.1003084 (PMC3521654; doi:10.1371/journal.ppat.1003084)
Supplement: Table S1 — The EBV infection assay. Primary B cells (5×10^4^) were infected with EBV or PBS (Mock) and subjected to an IF staining protocol using antibodies for EBNA2 (v-C20), NPM1 (C-19), or c-MYC at 0, 3 or 7 days after infection (dai) followed by a donkey anti-goat antibody conjugated to FITC (Green) or a goat anti-mouse antibody conjugated to rhodamine (red). Nuclei were counterstained with DAPI. The immunostained cells were quantified by flow cytometry. The summary data from immunostaining assays are shown. (PPT) [file ppat.1003084.s006.ppt]

## Slide 1
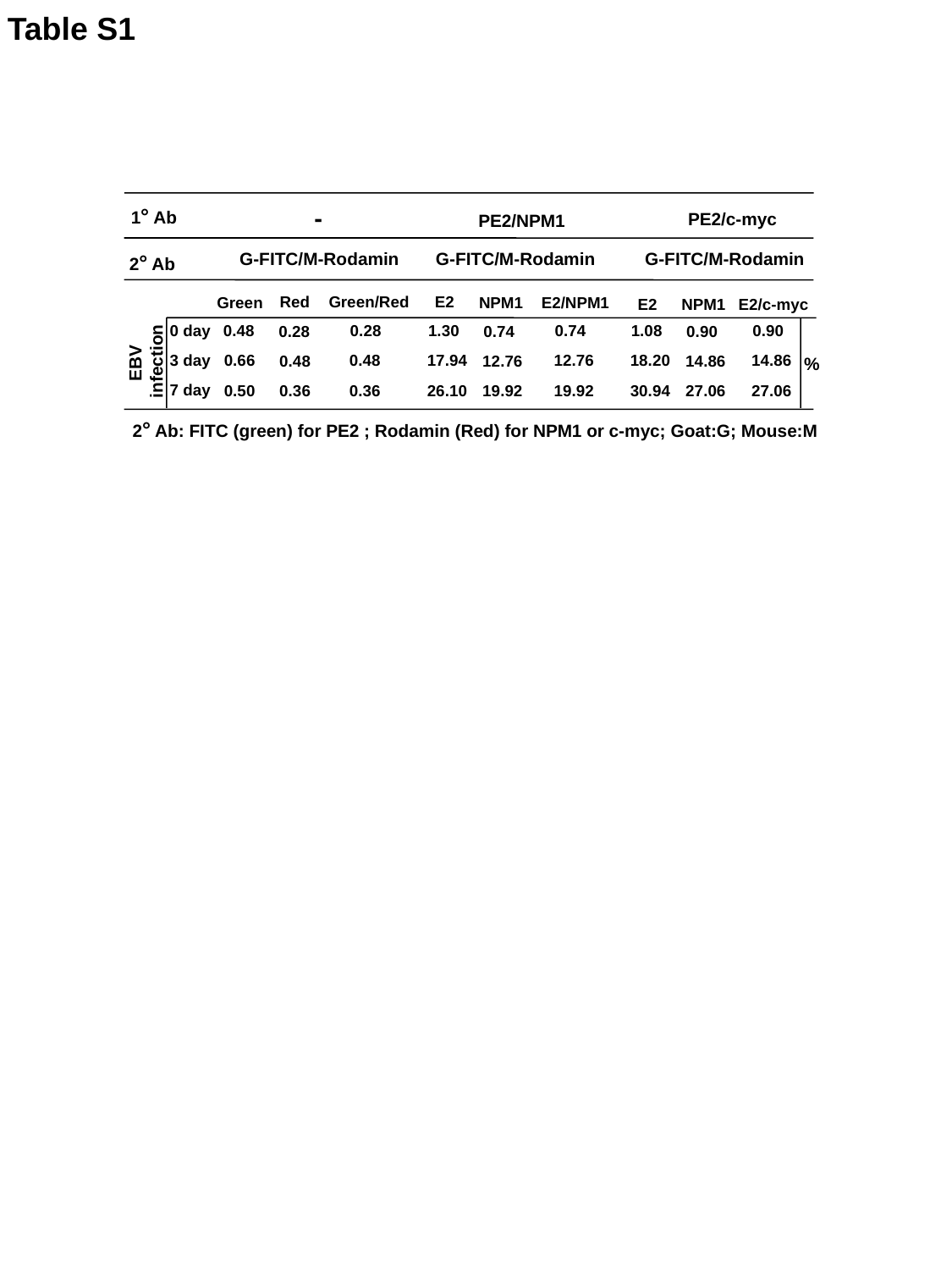

Table S1
-
1° Ab
PE2/c-myc
PE2/NPM1
G-FITC/M-Rodamin
G-FITC/M-Rodamin
G-FITC/M-Rodamin
2° Ab
Red
Green/Red
E2
Green
NPM1
E2/NPM1
NPM1
E2/c-myc
E2
0 day
0.28
0.74
0.90
0.48
1.30
1.08
0.28
0.74
0.90
EBV
infection
3 day
0.48
12.76
14.86
0.66
17.94
18.20
0.48
12.76
14.86
%
7 day
0.36
19.92
27.06
0.50
26.10
30.94
0.36
19.92
27.06
2° Ab: FITC (green) for PE2 ; Rodamin (Red) for NPM1 or c-myc; Goat:G; Mouse:M
